# Elevated BACH1 Contributes to Mitochondrial Succinylome Remodeling and Trophoblast Bioenergetic Dysfunction in Preeclampsia

**DOI:** 10.3390/antiox15070835

**Published:** 2026-07-01

**Authors:** Jiacheng Xu, Lujia Sun, Miaomiao Chen, Bingdi Chao, Jie He, Hongli Liu, Dongni Huang, Jie Wang, Lumei Xie, Philip N. Baker, Yubin Ding, Hongbo Qi, Xin Luo

**Affiliations:** 1 Department of Obstetrics, The First Affiliated Hospital of Chongqing Medical University, Chongqing 400016, China; xujiacheng@hospital.cqmu.edu.cn (J.X.); 2024320072@stu.cqmu.edu.cn (L.S.);; 2 Department of Obstetrics and Gynecology, Women and Children’s Hospital of Chongqing Medical University, Chongqing 401147, China; 3 Chongqing Key Laboratory of Maternal and Fetal Medicine, Chongqing Medical University, Chongqing 400016, China; 4 Joint International Research Laboratory of Reproduction and Development of the Chinese, Ministry of Education, Chongqing Medical University, Chongqing 400016, China; 5 Department of Obstetrics, Maternal and Child Health Hospital of Hubei Province, Huazhong University of Science and Technology, Wuhan 430070, China; 6 College of Life Sciences, University of Leicester, Leicester LE1 7RH, UK

**Keywords:** preeclampsia, trophoblast, BACH1, mitochondrial metabolism, lysine succinylation

## Abstract

Preeclampsia (PE) is a major pregnancy complication characterized by placental dysfunction and metabolic disturbances. Although mitochondrial abnormalities are frequently observed in PE, the upstream regulatory mechanisms remain incompletely understood. Here, we investigated the potential involvement of BACH1 in trophoblast dysfunction in PE and explored its association with mitochondrial metabolic alterations and protein succinylation. BACH1 expression was assessed in placental tissues and plasma samples from patients with PE, its functional effects were examined in trophoblast cell lines and BACH1 overexpression mouse models, and metabolic, bioenergetic, and succinylation-related alterations were evaluated using multi-omics and functional analyses. BACH1 expression was elevated in PE placentas and correlated with disease severity. In trophoblasts, BACH1 overexpression impaired proliferation, invasion, and trophoblast-mediated angiogenesis and was accompanied by mitochondrial and metabolic abnormalities, while quantitative succinylproteomic analysis revealed widespread alterations in mitochondrial protein succinylation. In vivo, BACH1 overexpression induced key PE-like features, including hypertension, fetal growth restriction, and placental abnormalities, and glycine supplementation partially rescued the trophoblast dysfunction associated with BACH1 overexpression. Together, evidence from clinical samples and experimental models suggests that BACH1 is associated with mitochondrial succinylation remodeling and trophoblast dysfunction in PE, supporting the hypothesis that BACH1-associated metabolic dysregulation and mitochondrial succinylation remodeling may contribute to PE pathogenesis. Further studies are required to establish the causal relevance and clinical significance of these mechanisms in human PE.

## 1. Introduction

Preeclampsia (PE), one of the “great obstetrical syndromes”, affects 3–5% of pregnancies worldwide, resulting in approximately 42,000 maternal deaths annually [1]. The diagnostic criteria include hypertension (systolic blood pressure ≥ 140 mmHg, diastolic blood pressure ≥ 90 mmHg, or both) accompanied by proteinuria or other organ dysfunction after 20 weeks of gestation [2]. PE poses significant health risks to both mothers and fetuses, increasing their susceptibility to long-term cardiovascular and metabolic diseases [3]. Despite the global burden, no effective therapeutic interventions currently exist, underscoring the need for a deeper understanding of its pathogenic mechanisms to guide future prevention and treatment strategies.

The pathogenesis of PE is commonly described by the “two-stage model” which posits that the disorder originates from defective placentation, primarily due to impaired function of extravillous trophoblasts (EVTs) [4,5]. This model has been refined to propose two distinct pathways: an extrinsic pathway featuring inadequate spiral artery remodeling (often in early-onset PE), and an intrinsic pathway related to a hyperactive placenta outgrowing uterine capacity (common in late-onset PE) [6,7]. Despite different origins, both pathways converge on inducing placental stress and dysfunction, ultimately driving the maternal syndrome.

Successful placentation is an energy-demanding process that requires precise metabolic adaptation of trophoblast cells. Mitochondria play a central role in supporting the bioenergetic and biosynthetic demands of trophoblast proliferation, differentiation, and invasion. In PE, a distinct metabolic shift in the placenta is observed, characterized by a downregulation of oxidative phosphorylation (OXPHOS) alongside a compensatory upregulation of glycolysis [8,9,10]. This shift, coupled with increased oxidative stress, results in bioenergetic insufficiency and is thought to be a key contributor to trophoblast dysfunction [11,12,13]. It should be noted that trophoblast metabolism is intrinsically heterogeneous, varying according to differentiation state, oxygen tension, gestational age, and experimental model [8,14]. Primary EVTs and immortalized trophoblast cell lines are not fully equivalent; primary EVTs exhibit more complex metabolic adaptations shaped by the in vivo placental microenvironment. Nevertheless, EVT-like cell lines such as HTR-8/SVneo share core metabolic requirements with primary EVTs, including coordinated mitochondrial respiration, TCA cycle activity, redox balance, and ATP production essential for proliferation, invasion, and vascular remodeling [15], making them widely used and reproducible systems for dissecting metabolic mechanisms relevant to EVT function [16,17,18]. In the present study, key findings obtained in HTR-8/SVneo cells were further validated in an independent trophoblast cell line (JEG-3) and in clinical placental tissue, and results are interpreted in conjunction with in vivo evidence throughout.

Cellular metabolic pathways are dynamically regulated not only at the transcriptional level but also through post-translational modifications (PTMs). Lysine succinylation (Ksucc) is a metabolically sensitive PTM closely linked to mitochondrial metabolism, TCA cycle activity, and intracellular succinyl-CoA availability. Recent studies have begun to reveal its placenta-specific relevance in hypertensive disorders of pregnancy. For example, SIRT5-mediated regulation of PRKAA2 succinylation has been reported to modulate apoptosis in human placental trophoblasts [19], whereas SIRT5-dependent desuccinylation of HOXB3 suppresses trophoblast proliferation, migration, and invasion in PE [20]. In addition, KAT2A-mediated succinylation of NLRP3 was shown to enhance NLRP3 stability and promote trophoblast pyroptosis in PE models [21]. These findings suggest that succinylation may participate in multiple aspects of trophoblast dysfunction, including apoptosis, impaired invasion, and inflammatory cell death. However, existing studies have mainly focused on individual substrates or single regulatory enzymes. Whether PE is associated with broader remodeling of the placental succinylome, particularly within mitochondrial metabolic pathways, remains insufficiently understood.

BACH1 (BTB and CNC homology 1) is a stress-responsive transcription factor best known for repressing antioxidant and heme metabolism pathways through competition with Nrf2. In addition to its established role in redox regulation, accumulating evidence indicates that BACH1 participates in the control of angiogenesis, mitochondrial biogenesis, cellular metabolism, and adaptation to hypoxic stress [22,23]. Emerging evidence further implicates BACH1 in placental biology. Dysregulated Bach1 expression has been observed in Hmox1-deficient placentas and is associated with altered hypoxia-responsive and angiogenic pathways [24]. More recently, BACH1 was shown to negatively regulate placental angiogenesis, at least in part through suppression of SLC25A51-mediated mitochondrial NAD^+^ transport, whereas genetic or pharmacological inhibition of BACH1 improved placental vascularization and fetal growth [25]. In trophoblast cells, BACH1 promotes oxidative stress-induced apoptosis through repression of the NRF2/HO-1 antioxidant pathway, highlighting its role in maintaining placental redox homeostasis [26]. Collectively, these findings suggest that BACH1 may function as a regulator of placental vascular function, mitochondrial metabolism, and oxidative stress adaptation. In the context of PE, aberrant oxidative stress, impaired angiogenesis, and mitochondrial dysfunction are recognized as central features of placental pathology. Consistent with this, differential expression of NRF2 and HO-1 has been reported in PE placentas [27,28]. Given that BACH1 functions as a key upstream repressor of the NRF2/HO-1 antioxidant axis and has been implicated in regulating mitochondrial metabolism and biogenesis in other biological contexts [29], it is plausible that dysregulated BACH1 signaling contributes to the metabolic and redox disturbances characteristic of PE. Notably, lysine succinylation is a mitochondrial metabolism-associated post-translational modification that is highly sensitive to changes in tricarboxylic acid cycle activity and succinyl-CoA availability. However, the expression status and pathological significance of BACH1 in PE placentas remain poorly defined, and whether BACH1 contributes to mitochondrial metabolic dysfunction and protein succinylation remodeling in PE has not been investigated.

Given the emerging roles of BACH1 in oxidative stress regulation and mitochondrial metabolism, we hypothesized that BACH1 may contribute to trophoblast dysfunction and metabolic abnormalities in PE. To test this possibility, we examined BACH1 expression in clinical PE samples and evaluated its functional effects in trophoblast cells and mouse models. We further investigated whether alterations in mitochondrial metabolism and protein succinylation are associated with trophoblast dysfunction induced by BACH1 overexpression. These studies were designed to explore a potential role for BACH1 in placental pathophysiology and to identify mechanisms that may contribute to PE development.

## 2. Materials and Methods

### 2.1. Data Collection

The microarray dataset GSE75010 was downloaded from the Gene Expression Omnibus (GEO) database [30] to evaluate BACH1 expression in placentas from patients with PE and normotensive controls. Processed expression data were log2-transformed and normalized prior to analysis, and expression distributions across samples were inspected for quality control. Differential expression analysis was performed using the limma package in R (v4.3.2). Linear models were fitted using the lmFit function, followed by empirical Bayes moderation using eBayes. *p* values were adjusted for multiple testing using the Benjamini–Hochberg method to control the false discovery rate (FDR). Normalized BACH1 expression values were subsequently extracted and compared between groups. Correlations between BACH1 and selected genes were evaluated using Pearson correlation analysis.

### 2.2. Clinical Sample Collection

This study was approved by the Ethics Committee of the First Affiliated Hospital of Chongqing Medical University, and written informed consent was obtained from all participants. This study included two independent cohorts, both restricted to early-onset PE. PE was diagnosed according to the American College of Obstetricians and Gynecologists (ACOG) guidelines [2], and early-onset PE was defined as PE diagnosed before 34 weeks of gestation. Patients with chronic hypertension, gestational diabetes, autoimmune disorders, infectious diseases, or other metabolic diseases were excluded from both cohorts. Cohort 1 (placental tissue cohort): Placental and decidual tissues were collected from women with normotensive pregnancies (*n* = 12) and patients diagnosed with PE (*n* = 12) at the time of cesarean section. Clinical characteristics are summarized in Table 1. Placental sampling was performed according to a standardized protocol [31]. Cohort 2 (NIPT plasma cohort): A separate cohort of maternal plasma samples collected for routine non-invasive prenatal testing (NIPT) at 12–14 weeks of gestation was retrospectively identified. These samples were subsequently stratified into women who later developed PE (*n* = 10) and women with uncomplicated pregnancies (*n* = 10). This cohort was used exclusively for early-pregnancy biomarker analyses. Clinical characteristics are summarized in Table 2.

### 2.3. Enzyme-Linked Immunosorbent Assay (ELISA)

Maternal plasma samples were collected in EDTA-containing tubes and centrifuged at 1000× *g* for 15 min at 4 °C. The supernatant was aliquoted and stored at −80 °C until analysis. BACH1 concentration was measured using the ELISA kit (MEIMIAN, Yancheng, China) according to the manufacturer’s instructions. All reagents and samples were equilibrated to room temperature for 30 min prior to use. A standard curve was generated using serial dilutions, with standard dilution buffer serving as the blank. Samples and standards were added to the pre-coated plate and incubated at 37 °C for 30 min. After five washes, enzyme-linked reagent was added, followed by another incubation at 37 °C for 30 min and five washes. Substrate solutions A and B were then added, incubated at 37 °C for 10 min, and the reaction was stopped. Absorbance was immediately read at 450 nm using a Multiskan GO spectrophotometer (Thermo Scientific, Waltham, MA, USA). Measurement precision was assessed by calculating coefficient of variation (*CV*) via the following formula: $CV=\left(\frac{Standard\;Deviation}{Mean\;Absorbance}\right)*100\%$.

### 2.4. Quantitative Real-Time PCR (qRT–PCR)

Total RNA was extracted from placental tissues or cells using TRIzol™ (Invitrogen, Carlsbad, CA, USA) according to the manufacturer’s instructions. RNA concentration and purity were assessed spectrophotometrically (NanoDrop 2000, Thermo Scientific, USA). cDNA was synthesized from 1.0 μg of total RNA using the PrimeScript™ RT Reagent Kit with gDNA Eraser (Takara, Shiga, Japan) on a Bio-Rad T100 Thermal Cycler (Bio-Rad Laboratories, Hercules, CA, USA) under the following conditions: 42 °C for 2 min (gDNA removal), 37 °C for 15 min (reverse transcription), and 85 °C for 5 min (enzyme inactivation).

qRT-PCR was performed using TB Green^®^ Premix Ex Taq™ II (Takara, Japan) on a CFX Connect Real-Time System (Bio-Rad, Hercules, CA, USA). The thermal cycling protocol consisted of an initial denaturation at 95 °C for 30 s, followed by 40 cycles of 95 °C for 5 s, 63.3 °C for 30 s, and 72 °C for 20 s. All reactions were run in triplicate. The primer sequences used in this study are listed in Table 3.

### 2.5. Western Blot Analysis

For Western blot analysis, placental tissue samples were randomly selected from the placental tissue cohort described above (7 of 12 samples per group). Total protein was extracted using RIPA lysis buffer (Beyotime, Shanghai, China) containing 1% PMSF and 1% phosphatase inhibitor. Protein concentration was determined with a BCA assay kit (Beyotime, Shanghai, China). Equal amounts of protein (20 μg per lane) were separated by SDS-PAGE at a constant voltage (100 V) and subsequently transferred onto PVDF membranes (Roche, Basel, Switzerland) using a wet transfer system at 4 °C for 2 h.

The membranes were blocked with 5% nonfat milk in TBST for 1 h at room temperature and then incubated overnight at 4 °C with specific primary antibodies. Target proteins included BACH1, cell cycle regulators (CDK2, Cyclin A2), lysine modification-related proteins (anti-succinyllysine, CPT1A, KAT2A, HAT1, SIRT5, SIRT7), and loading controls (α-Tubulin, β-actin, GAPDH). All primary antibodies were used at the dilutions recommended by the manufacturers. Complete details regarding catalog numbers, and manufacturers are provided in Supplementary Table S1.

Following incubation, the membranes were washed with TBST and incubated for 1 h at room temperature with HRP-conjugated goat anti-mouse (ZB-2305, ZSGB-BIO, Beijing, China) or goat anti-rabbit (ZB-2301, ZSGB-BIO, Beijing, China) secondary antibodies. Protein bands were visualized using WesternBright™ ECL substrate (Advansta, Menlo Park, CA, USA) and quantified with Quantity One software (v4.6.9, Bio-Rad Laboratories, Hercules, CA, USA).

### 2.6. Immunofluorescence

Immunofluorescence was performed to examine the expression and localization of BACH1 in human placental/decidual tissues, mouse placental tissue, primary EVTs, and the HTR-8/SVneo trophoblast cell line.

Frozen sections of human and mouse tissues were equilibrated at room temperature for 30 min. Sections were washed in TBST for 5 min to remove cryoprotectant, permeabilized with 0.2% Triton X-100 (Beyotime, Shanghai, China) for 15 min at room temperature, and then washed three times with PBS. Nonspecific sites were blocked with 10% goat serum (Boster, Pleasanton, CA, USA) for 1 h at room temperature. The sections were incubated overnight at 4 °C with the following primary antibodies: rabbit anti-BACH1 (1:200, 14018-1-AP, Proteintech, Rosemont, IL, USA), mouse anti-HLA-G (1:200, 66447-1-Ig, Proteintech, Rosemont, IL, USA), and mouse anti-CK7 (1:200, GB12225, Servicebio, Wuhan, China). After washing with PBS, sections were incubated with Alexa Fluor 488-conjugated goat anti-rabbit or Alexa Fluor 594-conjugated goat anti-mouse secondary antibodies (1:200, Proteintech, Rosemont, IL, USA) for 1 h at 37 °C in the dark. Nuclei were counterstained with DAPI (Beyotime, Shanghai, China), and sections were mounted with antifade medium.

Primary EVTs and HTR-8/SVneo cells were seeded in 48-well plates. After adherence, cells were fixed with 4% paraformaldehyde for 30 min at room temperature. Subsequent steps for permeabilization, blocking, antibody incubation, and nuclear staining were identical to those described for tissue sections above.

All images were captured using an EVOS FL Auto 2 microscope (Thermo Fisher Scientific, Waltham, MA, USA). The fluorescence intensity of specific signals was quantified from at least three independent fields per sample using ImageJ software (National Institutes of Health).

### 2.7. Animal Experiments

All animal procedures were approved by the Institutional Animal Care and Use Committee of Chongqing Medical University (Approval No. IACUC-CQMU-2023-0411) and conducted in accordance with national guidelines. The animals were housed and experiments were performed at the Experimental Animal Center of Chongqing Medical University, where they were maintained under controlled temperature (22 ± 2 °C) and humidity (50 ± 10%) with a 12-h light/dark cycle and free access to food and water.

At the end of the experiments, all animals were deeply anaesthetized by inhalation of isoflurane (3–5% in oxygen at a flow rate of 0.5–1 L/min) in an induction chamber. Once deep anaesthesia was confirmed by the absence of the pedal withdrawal reflex, the animals were euthanised by cervical dislocation. Death was confirmed by respiratory arrest.

### 2.8. Animals and Adenovirus-Mediated Placental Overexpression Model

Institute of Cancer Research (ICR) mice (8–12 weeks old) were obtained from the Experimental Animal Center of Chongqing Medical University. Mice were mated overnight, and the presence of a vaginal plug the following morning was designated as gestational day (GD) 0.5. Pregnant mice were randomly assigned to experimental groups using a computer-generated randomization schedule. The investigators responsible for histological evaluation, immunostaining quantification, and image analysis were blinded to group allocation until completion of data analysis.

Pregnant mice were randomly allocated into two groups: one receiving a control adenovirus (Ad-Ctrl, *n* = 10) and the other receiving an adenovirus overexpressing mouse Bach1 (Ad-Bach1, *n* = 10). Both adenoviruses (pAdEasy-EF1-Bach1-3flag-CMV-EGFP and its control) were purchased from Hanheng Biotechnology Co., Ltd, Shanghai, China. On GD 8.5, mice received a single intravenous injection via the tail vein of 2 × 10^9^ plaque-forming units (PFU) of the respective adenovirus in 100 μL. Subsets of mice were euthanized at GD 14.5 (*n* = 3 per group) and GD 18.5 (*n* = 7 per group) for tissue collection.

### 2.9. Synthesis and Characterization of Trophoblast-Targeted Nanoparticles (NPs)

Trophoblast-targeted NPs were synthesized using lipid–polymer hybrid technology as previously described [32]. Briefly, NPs were prepared via a single-step sonication method using poly(lactic-co-glycolide) (PLGA), soybean lecithin, and DSPE-PEG-COOH. The placental chondroitin sulfate A-binding peptide (plCSA-BP) was conjugated to the NP surface via EDC/NHS chemistry. The resulting NPs were loaded with either a control plasmid (plCSA-vector) or a BACH1 overexpression plasmid (plCSA-Bach1), both of which were obtained from Hanheng Biotechnology Co., Ltd. The hydrodynamic size, polydispersity index (PDI), and zeta potential of the synthesized NPs were measured at room temperature using a Delsa™ Nano C particle analyzer (Beckman Coulter, Brea, CA, USA).

### 2.10. Trophoblast-Specific BACH1 Overexpression and Targeting

A separate cohort of pregnant ICR mice was used for the targeted delivery model. Pregnant mice were randomly assigned to either the plCSA-vector group (*n* = 5) or the plCSA-Bach1 group (*n* = 6). Investigators performing histological assessment and quantitative image analyses were blinded to treatment allocation. Mice received intravenous injections (100 µL tail-vein) of either plCSA-vector NPs or plCSA-Bach1 NPs on GD 8.5, 10.5, and 12.5. The concentration of the Bach1 plasmid in the formulation was approximately 0.40 mg/mL. All mice were euthanized at GD 18.5 for tissue collection.

To validate the targeting specificity, a separate group of pregnant mice at GD 18.5 received a single injection of plCSA-BP-conjugated NPs loaded with indocyanine green (ICG). Thirty minutes post-injection, the in vivo biodistribution of fluorescence signals was assessed using an OI600 imaging system (BIO-OI, Shanghai, China).

### 2.11. Hematoxylin and Eosin (H&E) Staining

Freshly harvested mouse placental and renal tissues were fixed in 4% paraformaldehyde, embedded in paraffin, and sectioned at a thickness of 3 μm. For H&E staining, sections were first deparaffinized in xylene (twice for 15 min each) and rehydrated through a graded ethanol series (100%, 90%, 80%, 70%; 2 min each). After a brief rinse in distilled water, sections were stained with hematoxylin for 3 min, rinsed, differentiated in 1% acid alcohol for 3–5 s, and rinsed again in running tap water for 20 s. Counterstaining was performed with eosin for 15–30 s. Subsequently, sections were dehydrated through an ascending ethanol series (70%, 80%, 90%, 100%; 10 s each), cleared in xylene for 5 min, and mounted with neutral resin. Stained sections were examined and imaged under an optical microscope (Life Technologies, Carlsbad, CA, USA). Morphometric analysis was performed using ImageJ software (version 1.53o, National Institutes of Health, Bethesda, MD, USA; https://imagej.nih.gov/ij/, accessed on 1 May 2026).

### 2.12. Immunohistochemistry (IHC)

Placental tissues from mice were fixed in 4% paraformaldehyde, embedded in paraffin, and sectioned at a thickness of 4–5 μm. For immunohistochemical staining, sections were first baked at 60 °C for 2 h, deparaffinized in xylene (twice, 15 min each), and rehydrated through a graded ethanol series (100%, 90%, 80%, 70%; 5 min each).

Antigen retrieval was performed by microwave heating in 10 mM sodium citrate buffer (pH 6.0) for 15 min. Endogenous peroxidase activity was quenched by incubating sections with 3% hydrogen peroxide for 10 min at room temperature. After washing with PBS, sections were blocked with 10% normal goat serum for 1 h at room temperature.

Sections were then incubated overnight at 4 °C with a mouse anti-CK7 primary antibody (1:200, EPR17078, Abcam, Cambridge, UK). After thorough washing, sections were incubated with a horseradish peroxidase (HRP)-conjugated goat anti-mouse secondary antibody (1:200) for 30 min at room temperature. Signal was developed using a 2-amino-9-ethylcarbazole (AEC) substrate kit, followed by counterstaining with hematoxylin.

### 2.13. Blood Pressure Measurement

Mouse systolic blood pressure was measured using a non-invasive tail-cuff system (BP-2000, Visitech Systems, Sunderland, UK). Prior to formal measurement, all mice were acclimated to the restraint device and measurement environment for 3 consecutive days. For each measurement session, mice were gently restrained on a temperature-controlled platform (maintained at 36 °C) with their tails placed in the sensor cuffs. After a 5-min stabilization period, systolic blood pressure was recorded in accordance with the manufacturer’s protocol. Five consecutive readings were obtained per mouse per session, and the mean value was calculated for statistical analysis. The total restraint time for each mouse did not exceed 30 min.

### 2.14. Isolation and Culture of Primary EVTs

HLA-G^+^ primary EVTs were isolated from first-trimester (6–9 weeks) human villous tissue following an established protocol [33]. Briefly, fresh tissue was washed with Mg^2+^/Ca^2+^-free HBSS (Gibco, Waltham, MA, USA), dissected into 1–2 mm fragments, and digested in 0.25% trypsin (Gibco) supplemented with 1.25 mg/mL DNase I (Sigma-Aldrich, St. Louis, MO, USA) at 37 °C for 10 min. The digestion was terminated by adding an equal volume of culture medium containing 10% fetal bovine serum (FBS). The cell suspension was filtered through a 100 μm cell strainer, and the isolated EVTs were seeded onto fibronectin-coated 48-well plates. Cells were maintained in EVT medium (DMEM/F12 supplemented with 10% FBS and 0.05 mg/mL gentamicin) at 37 °C in a humidified atmosphere of 5% CO_2_ and used for subsequent immunofluorescence experiments.

### 2.15. Cell Lines

The human trophoblast cell line HTR-8/SVneo (ATCC^®^ CRL-3271™, Manassas, VA, USA) was cultured in RPMI 1640 medium (Gibco, Waltham, MA, USA). The human choriocarcinoma cell line JEG-3 (ATCC^®^ HTB-36™) was cultured in Eagle’s Minimum Essential Medium (EMEM, Gibco, Waltham, MA, USA). Both media were supplemented with 10% FBS (PAN-Biotech, Aidenbach, Germany) and 1% penicillin–streptomycin (Gibco, Waltham, MA, USA). All cells were maintained at 37 °C in a 5% CO_2_ incubator and were routinely passaged. Cells between passages 5 and 10 were used for experiments.

### 2.16. Lentivirus-Mediated BACH1 Overexpression

Lentiviruses carrying the human BACH1 overexpression construct (Ubi-MCS-3FLAG-CBh-gcGFP-IRES-puromycin) and the corresponding control vector were purchased from GeneChem (Shanghai, China). Target cells at approximately 30% confluence were transduced with lentivirus at a multiplicity of infection (MOI) of 30. After 72 h, transduction efficiency was estimated by observing green fluorescent protein (GFP) expression under a fluorescence microscope. To establish stable cell lines, transduced cells were selected and maintained in culture medium containing 2 μg/mL puromycin.

### 2.17. siRNA-Mediated BACH1 Knockdown

A small interfering RNA (siRNA) targeting human BACH1 (si-BACH1: sense 5′-GCAAUGGAACCUGAAGAAATT-3′) and a negative control siRNA (si-NC) were synthesized by GenePharma (Shanghai, China). For transfection, HTR-8/SVneo cells were seeded in 6-well plates at a density of 2 × 10^5^ cells per well and grown to 70–90% confluence. Cells were then transfected with 100 pmol of siRNA using Lipofectamine 2000 reagent (Invitrogen, Carlsbad, CA, USA) according to the manufacturer’s instructions. Cells were harvested for subsequent RNA or protein analysis 24 to 48 h post-transfection.

### 2.18. Tube Formation Assay

To assess the angiogenic potential of trophoblasts, a tube formation assay was performed using HTR-8/SVneo cells as previously described [34]. Matrigel (BD Biosciences, San Jose, CA, USA) was thawed overnight at 4 °C. Then, 50 μL of Matrigel was coated onto each well of a 48-well plate and allowed to polymerize at 37 °C for 30 min. HTR-8/SVneo cells were trypsinized, resuspended in complete culture medium, and seeded onto the polymerized Matrigel at a density of 2 × 10^4^ cells per well. After 4–6 h of incubation, tube-like structures were observed. Representative bright-field images from at least three independent wells per group were captured using IX73 inverted microscope (Olympus, Tokyo, Japan) The formation of capillary-like networks was quantified using the Angiogenesis Analyzer tool [35] for ImageJ software.

### 2.19. Transwell Assay

Cell invasion ability was assessed using 24-well Transwell chambers with an 8.0 μm pore size (Corning, NY, USA). Matrigel (BD Biosciences, San Jose, CA, USA) was diluted to 1 mg/mL in ice-cold, serum-free medium. Then, 60 µL of the diluted Matrigel was carefully added to the upper chamber of each insert and allowed to polymerize at 37 °C for 4 h. Subsequently, HTR-8/SVneo cells (5 × 10^4^ cells per well) resuspended in serum-free medium were seeded into the Matrigel-coated upper chamber. The lower chamber was filled with 500 µL of complete culture medium containing 10% FBS as a chemoattractant. After incubation for 24 h at 37 °C in a 5% CO_2_ incubator, non-invading cells on the upper surface of the membrane were gently removed with a cotton swab. Cells that had invaded through the Matrigel and membrane were fixed with 4% paraformaldehyde for 15 min, stained with 0.1% crystal violet for 20 min, and rinsed with PBS. Membranes were then excised and mounted on glass slides. Invaded cells were imaged using an EVOS FL Color Imaging System (Thermo Fisher Scientific, Waltham, MA, USA). The number of invaded cells was counted manually using ImageJ software (version 1.53o, National Institutes of Health, Bethesda, MD, USA; https://imagej.nih.gov/ij/, accessed on 1 May 2026).

### 2.20. 5-ethynyl-2′-deoxyuridine (EdU) Staining

Proliferation of HTR-8/SVneo cells was evaluated using the Cell-Light™ EdU Apollo567 In Vitro Kit (RiboBio, Guangzhou, China) according to the manufacturer’s protocol. Cells were seeded in 96-well plates at a density of 8 × 10^3^ cells per well and cultured overnight to allow adherence. EdU was added to the culture medium at a final concentration of 50 µM, and cells were incubated for 2 h at 37 °C. Subsequently, cells were fixed with 4% paraformaldehyde for 30 min at room temperature, neutralized with 2 mg/mL glycine for 5 min, and permeabilized with 0.5% Triton X-100 for 10 min. For detection, Apollo^®^ 567 staining solution was added to each well and incubated for 30 min at room temperature in the dark. Cell nuclei were then counterstained with DAPI (Beyotime, Shanghai, China) for 10 min. Images were acquired using an EVOS FL Auto 2 fluorescence microscope (Thermo Fisher Scientific, Waltham, MA, USA). The numbers of EdU-positive (red) and total DAPI-positive (blue) nuclei were quantified using ImageJ software (version 1.53o). The proliferation rate was calculated as the percentage of EdU-positive cells relative to the total number of DAPI-positive cells in the same field.

### 2.21. Cell Viability Assay

Cell viability was assessed using a Cell Counting Kit-8 (CCK-8, Monmouth Junction, NJ, USA). HTR-8/SVneo cells were seeded in 96-well plates at a density of 5 × 10^3^ cells/well in 100 µL of complete culture medium and incubated overnight at 37 °C in a 5% CO_2_ incubator to allow adherence. After the designated treatments, 10 µL of CCK-8 reagent was carefully added to each well to avoid introducing bubbles. The plate was gently shaken to ensure mixing and then incubated at 37 °C for 1 to 4 h, protected from light. The absorbance of each well was measured at 450 nm using a Multiskan GO microplate reader (Thermo Fisher Scientific, Waltham, MA, USA).

### 2.22. Cell Cycle Analysis

The cell cycle distribution of HTR-8/SVneo cells was analyzed using a Cell Cycle Assay Kit (Elabscience, Wuhan, China) and flow cytometry. Cells were harvested, washed twice with PBS, and centrifuged at 1000 rpm for 5 min. The cell pellet was resuspended in 100 μL of PBS, and fixation was performed by the dropwise addition of 900 µL of ice-cold 75% ethanol with gentle vortexing. Prior to staining, cells were centrifuged at 1000 rpm for 5 min, the ethanol was carefully removed, and the pellet was washed twice with PBS. Cells were then treated with 100 µL of RNase A solution and incubated at 37 °C for 30 min. Subsequently, 400 µL of propidium iodide (PI) staining solution (50 µg/mL) was added, and samples were incubated in the dark at 4 °C for 30 min. PI fluorescence was measured using a CytoFLEX flow cytometer (Beckman Coulter, Brea, CA, USA) with an excitation wavelength of 488 nm and detection in the PE channel. A minimum of 10,000 events were collected per sample. Cell cycle distribution (G0/G1, S, and G2/M phases) was analyzed using CytExpert software (v2.4, Beckman Coulter, Brea, CA, USA).

### 2.23. Metabolomic Profiling by Gas Chromatography–Mass Spectrometry (GC–MS)

Untargeted metabolomics analysis of HTR-8/SVneo cells after BACH1 modulation was performed by GC-MS. Cells were quenched with liquid nitrogen, and metabolites were extracted with 1.5 mL methanol: chloroform (9:1, *v*/*v*) after adding 0.3 μmol d_4_-alanine as an internal standard. The extract was vortexed, centrifuged (15 min, 4 °C, max speed), and the supernatant was dried in a Labconco CentriVap^®^ concentrator (Labconco Corporation, Kansas City, MO, USA).

Derivatization was carried out via methyl chloroformate (MCF) following the methods of Smart et al. [36]. Dried samples were treated sequentially with NaOH, methanol, pyridine, and MCF, vortexed twice, then extracted into chloroform. The organic layer was dried over anhydrous sodium sulfate and transferred to GC vials.

Analysis was performed on an Agilent 7890GC/5975MSD system (Agilent Technologies, Santa Clara, CA, USA) equipped with a ZB-1701 capillary column (Phenomenex, Torrance, CA, USA). Helium carrier gas flow was 1 mL/min in pulsed splitless mode (injector 290 °C). Mass spectra were acquired at 1.562 scans/s with a 5.5-min solvent delay.

Metabolites were deconvoluted using AMDIS and identified against an in-house MCF library (match ≥ 85%, retention time ± 1 min). Relative abundances were normalized to the internal standard and total ion current using MassOmics. Differential metabolites were identified via the BioDeep platform with thresholds of VIP > 1 and *p* < 0.05.

### 2.24. Mitochondrial Membrane Potential Detection

The mitochondrial membrane potential (ΔΨm) in HTR-8/SVneo cells was assessed using the fluorescent probe TMRM (TMRM; Invitrogen, Carlsbad, CA, USA). Cells were incubated with 100 nM TMRM in complete medium at 37 °C for 30 min in the dark. After incubation, cells were washed twice with warm PBS, trypsinized, and resuspended in PBS containing 2% fetal bovine serum. Fluorescence intensity was immediately measured using a CytoFLEX flow cytometer (Beckman Coulter, Brea, CA, USA). For each sample, at least 10,000 events were collected. The mean fluorescence intensity (MFI) of the TMRM signal (excitation 488 nm, emission 574 nm) was analyzed using CytExpert software (Beckman Coulter, Brea, CA, USA) and used as an indicator of ΔΨm.

### 2.25. Analysis of Cellular Mitochondrial Bioenergetics

Mitochondrial bioenergetics in HTR-8/SVneo cells were assessed using a Seahorse XFe24 Analyzer (Agilent Technologies, Santa Clara, CA, USA) to evaluate the effects of BACH1 on cellular metabolism.

Cells were seeded into Seahorse XF24 cell culture microplates (Agilent Technologies, Santa Clara, CA, USA) at a density of 2 × 10^4^ cells per well and incubated overnight at 37 °C in a 5% CO_2_ humidified incubator to reach 80–90% confluence. Each experimental condition was analyzed using five technical replicates, and all experiments were independently repeated at least three times. On the day of the assay, the growth medium was replaced with pre-warmed Seahorse XF RPMI Medium (pH 7.4, Agilent Technologies, Santa Clara, CA, USA), and cells were incubated in a non-CO_2_ incubator at 37 °C for 60 min to allow temperature and pH equilibration. For the Mitochondrial Stress Test, the assay medium was supplemented with 10 mM glucose, 1 mM sodium pyruvate, and 2 mM L-glutamine. For the Glycolysis Stress Test, only 2 mM L-glutamine was added to the base medium.

Following the manufacturer’s protocols, sensor cartridges were hydrated overnight and loaded with the appropriate metabolic modulators. For the Mitochondrial Stress Test, injection ports contained: port A, 1.5 µM oligomycin; port B, 1.0 µM carbonyl cyanide-4-(trifluoromethoxy)phenylhydrazone (FCCP); and port C, 0.5 µM rotenone plus 0.5 µM antimycin A. For the Glycolysis Stress Test, injection ports contained: port A, 10 mM glucose; port B, 1.0 µM oligomycin; and port C, 50 mM 2-deoxy-D-glucose (2-DG).

The oxygen consumption rate (OCR) and extracellular acidification rate (ECAR) were measured in real time. For each measurement point, data were acquired over three measurement cycles consisting of mixing, equilibration, and measurement phases according to the manufacturer’s standard protocol. Background correction was performed using cell-free wells. Raw OCR and ECAR data were analyzed using Wave software (v2.x, Agilent Technologies, Santa Clara, CA, USA).

Following the assay, cells were lysed, and the total protein content in each well was quantified using a BCA assay kit (Beyotime, Shanghai, China). All OCR and ECAR values were normalized to the total protein amount and are expressed as pmol O_2_/min/µg protein and mpH/min/µg protein, respectively.

### 2.26. Succinyllysine Proteomic Analysis (4D LC-MS/MS)

Quantitative succinylproteomic analysis was performed to assess the effect of BACH1 overexpression on protein lysine succinylation (Ksucc). HTR-8/SVneo cells transfected with control vector (Vector) or BACH1 overexpression plasmid (OE-BACH1) were collected, washed with ice-cold PBS, and flash-frozen in liquid nitrogen. Protein extraction, tryptic digestion, enrichment of succinylated peptides, and mass spectrometry were performed by PTM Bio (Hangzhou, China).

Raw data were searched in MaxQuant (v1.6.15.0) against the UniProt human database (20,395 sequences) with a target-decoy and contaminant database. The FDR for identification was controlled at 1% at the spectrum (PSM), peptide, and protein levels, with each protein requiring at least one unique peptide. To distinguish succinylation changes from protein-abundance changes, global proteomic and succinylproteomic analyses were performed in parallel, and each succinylation site was normalized to its corresponding protein abundance; all downstream analyses used these protein-normalized values.

Differentially succinylated sites were defined by FC > 1.5 or <1/1.5 and *p* < 0.05 (two-tailed Student’s *t*-test on Log2-transformed data) and subjected to GO, KEGG, protein domain, and protein–protein interaction analyses.

### 2.27. Statistical Analysis

All statistical analyses were performed using GraphPad Prism software (version 8.0, GraphPad Software, San Diego, CA, USA). Data are presented as the mean ± standard error of the mean (SEM). Normality of distribution was assessed using the Kolmogorov–Smirnov test. For comparisons between two groups, Student’s *t*-test was used for data meeting assumptions of normality and homogeneity of variance; Welch’s correction was applied when variances were unequal. For non-normally distributed data, the Mann–Whitney U test was employed. For comparisons among multiple groups, one-way analysis of variance (ANOVA) was used for parametric data, followed by appropriate post hoc tests. Welch’s ANOVA or the Kruskal–Wallis test was applied for data that did not meet the assumptions of normality or homogeneity of variance. Statistical significance is denoted as follows: ns, not significant; * *p* < 0.05; ** *p* < 0.01; *** *p* < 0.001; **** *p* < 0.0001.

## 3. Results

### 3.1. BACH1 Is Upregulated in Preeclamptic Placentas and Correlates with Clinical Severity

**Figure 1:**
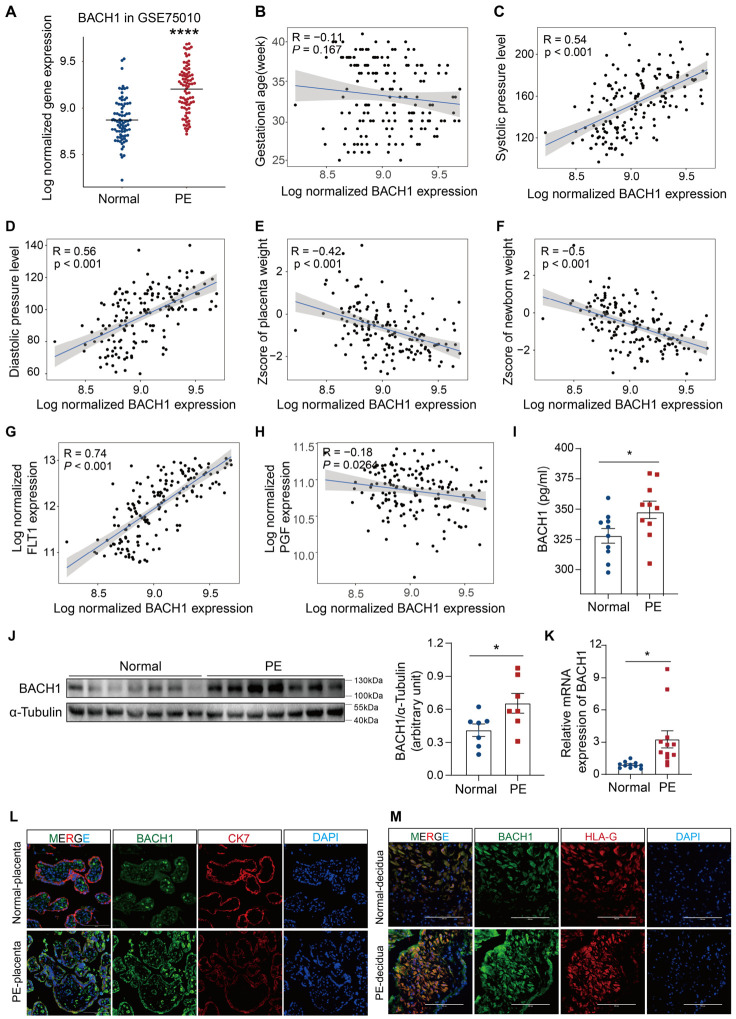
Elevated BACH1 expression is associated with PE: (**A**) Differential expression of BACH1 mRNA in placental tissues from the GSE75010 dataset (Normal, *n* = 77; PE, *n* = 80). (**B**) Pearson correlation analysis between BACH1 expression and gestational age shows no significant association. (**C**–**H**) Pearson correlation analyses of BACH1 expression with clinical parameters: a positive correlation with systolic blood pressure (**C**) and diastolic blood pressure (**D**), and negative correlations with placental weight (**E**), newborn weight (**F**), and placental growth factor (PlGF) levels (**H**); a positive correlation with fms-related tyrosine kinase 1 (FLT1) levels (**G**) is shown. In the correlation plots, each dot represents an individual sample, the solid line indicates the linear regression fit, and the shaded area denotes the 95% confidence interval. (**I**) Enzyme-linked immunosorbent assay (ELISA) quantification of BACH1 protein levels in first-trimester maternal plasma (Normal, *n* = 10; PE, *n* = 10). (**J**) Representative Western blot (**left**) and quantification (**right**) of BACH1 protein in term placental tissues (Normal, *n* = 7; PE, *n* = 7). α-Tubulin served as the loading control. (**K**) Quantitative real-time polymerase chain reaction (qRT-PCR) analysis of BACH1 mRNA in term placental tissues (Control, *n* = 11; PE, *n* = 12). (**L**) Representative immunofluorescence images showing BACH1 (green) co-localization with the trophoblast marker CK7 (red) in term placental villi from normal and PE pregnancies. Nuclei are stained with DAPI (blue). Scale bars, 100 μm. (**M**) Representative immunofluorescence images showing BACH1 (green) co-localization with the extravillous trophoblast marker HLA-G (red) in term decidua from normal and PE pregnancies. Nuclei are stained with DAPI (blue). Scale bars, 200 μm. Data were analyzed by unpaired two-tailed Student’s *t* test. Results are presented as mean ± SEM. * *p* < 0.05, **** *p* < 0.0001.

To investigate the potential involvement of BACH1 in PE, we first analyzed its expression in a public placental transcriptome dataset (GSE75010). BACH1 mRNA levels were significantly elevated in placentas from PE patients compared to healthy controls (Figure 1A). This upregulation was independent of gestational age (Figure 1B) but demonstrated strong clinical relevance: it correlated positively with maternal systolic blood pressure (Figure 1C) and diastolic blood pressure (Figure 1D), and negatively with placental weight (Figure 1E) and neonatal birth weight (Figure 1F). Furthermore, BACH1 expression exhibited a strong positive correlation with FLT1 (Figure 1G), a key anti-angiogenic factor in PE, and a negative correlation with PlGF (Figure 1H). These bioinformatic findings positioned BACH1 as a molecule closely associated with the clinical hallmarks of PE.

We next validated these findings in our clinical cohorts. In maternal plasma collected at 12–14 weeks of gestation, prior to clinical onset, BACH1 protein levels were already higher in women who later developed PE (Figure 1I). At term, placentas from PE patients consistently showed increased BACH1 expression at both the protein and mRNA levels compared to normal controls (Figure 1J,K).

To pinpoint the cellular sources of BACH1 at the maternal-fetal interface, we performed multiplex immunofluorescence. BACH1 co-localized with the trophoblast marker CK7 and the EVT marker HLA-G, indicating its expression in both syncytiotrophoblasts and EVTs (Figure 1L,M).

Collectively, these results demonstrate that BACH1 is aberrantly upregulated in PE placentas from early to late gestation. Its strong association with clinical severity parameters and angiogenic imbalance highlights a potential involvement of BACH1 in PE pathophysiology and warrants further investigation into its functional role in trophoblast biology.

### 3.2. Placental Bach1 Overexpression Recapitulates Key Features of PE in Mice

**Figure 2:**
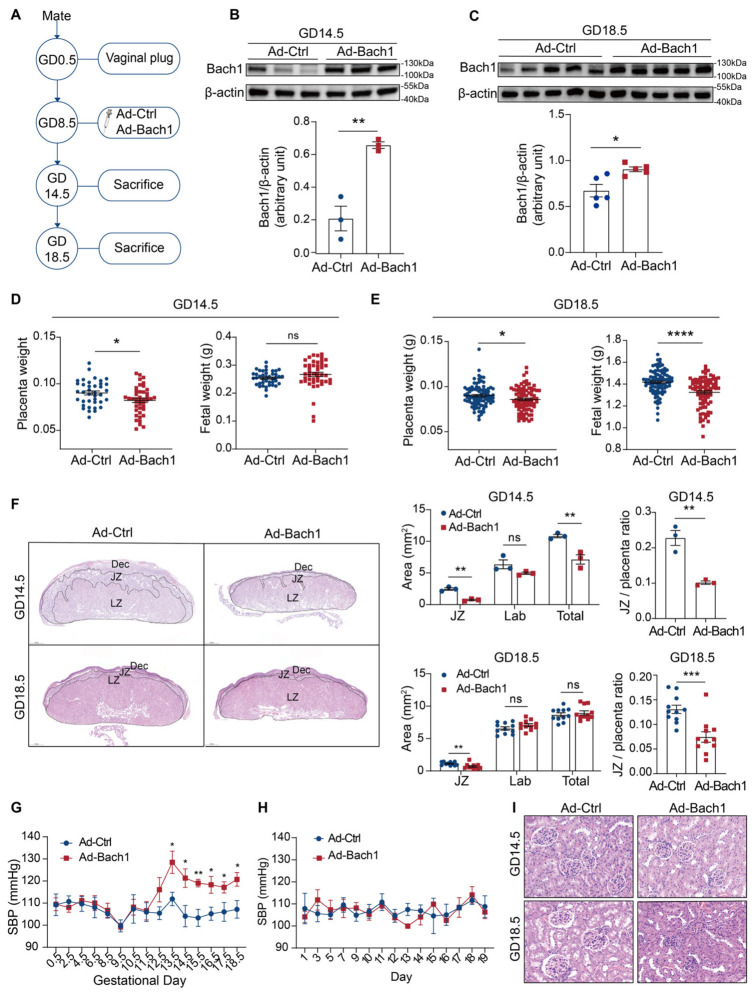
Overexpression of Bach1 in mouse placental tissue induces a preeclampsia-like phenotype: (**A**) Schematic diagram of the experimental protocol for adenovirus-mediated Bach1 overexpression in pregnant mice. (**B**,**C**) Representative Western blot (**left**) and quantification (**right**) of Bach1 protein expression in placental tissues at gestational day (GD) 14.5 (Ad-Ctrl, *n* = 3; Ad-Bach1, *n* = 3) and GD 18.5 (Ad-Ctrl, *n* = 5; Ad-Bach1, *n* = 5). β-actin served as the loading control. (**D**,**E**) Placental weight and fetal weight at GD 14.5 (**D**) and GD 18.5 (**E**). (**F**) Representative hematoxylin and eosin (H&E)-stained images of placental at GD 14.5 and GD 18.5 (**left**). Statistical analysis of the corresponding zone areas is shown on the right. Lab, labyrinth zone; JZ, junctional zone; Dec, decidua zone. Scale bar, 500 μm. (**G**,**H**) Systolic blood pressure monitored from GD 0.5 to GD 18.5 in pregnant mice ((**G**); Ad-Ctrl, *n* = 6; Ad-Bach1, *n* = 6) and over the corresponding period in non-pregnant mice ((**H**); Ad-Ctrl, *n* = 3; Ad-Bach1, *n* = 3). (**I**) Representative H&E-stained images of kidney sections from pregnant mice at GD 14.5 and GD 18.5. Scale bar, 20 μm. Data are presented as mean ± SEM. Statistical significance was determined by unpaired two-tailed Student’s *t* test. * *p* < 0.05, ** *p* < 0.01, *** *p* < 0.001, **** *p* < 0.0001 compared to the Ad-Ctrl group at the corresponding time point. ns, not significant.

To investigate whether elevated Bach1 contributes to PE-associated phenotypes rather than simply representing a secondary consequence of disease progression, we generated a placental overexpression model by intravenously injecting pregnant mice with Bach1 (mouse homolog of human BACH1)-encoding adenovirus (Ad-Bach1) or control virus (Ad-Ctrl) on gestational day (GD) 8.5 (Figure 2A). Successful placental transduction was confirmed by widespread EGFP fluorescence in placentas (Figure S1) and by elevated Bach1 protein levels at GD 14.5 and GD 18.5 (Figure 2B,C).

Bach1 overexpression impaired placentation and fetal growth. Placental weight was significantly reduced at GD 14.5 (Figure 2D), and both placental and fetal weights were decreased at GD 18.5 compared to controls (Figure 2E). Histological analysis revealed that at GD 14.5, both total placental area and the junctional zone were significantly smaller in Ad-Bach1 mice. By GD 18.5, while total area showed no difference, the junctional zone remained underdeveloped, indicating a persistent defect in this key compartment (Figure 2F).

Concurrently, pregnant mice overexpressing Bach1 exhibited maternal manifestations resembling those observed in PE. Systolic blood pressure increased significantly from GD 13.5 onward (Figure 2G), an effect specific to pregnancy as it was absent in non-pregnant mice (Figure 2H). At GD 18.5, these mice also exhibited glomerular constriction (Figure 2I), mirroring renal pathology seen in human PE.

In summary, placental Bach1 overexpression was associated with the development of multiple PE-like features, encompassing fetal growth restriction, defective placental morphogenesis, maternal hypertension, and end-organ damage. These findings suggest that elevated placental Bach1 is sufficient to recapitulate several PE-associated features in mice and support its potential involvement in PE pathogenesis.

### 3.3. BACH1 Impairs Trophoblast Function by Inhibiting Proliferation, Invasion, and Angiogenic Potential

**Figure 3:**
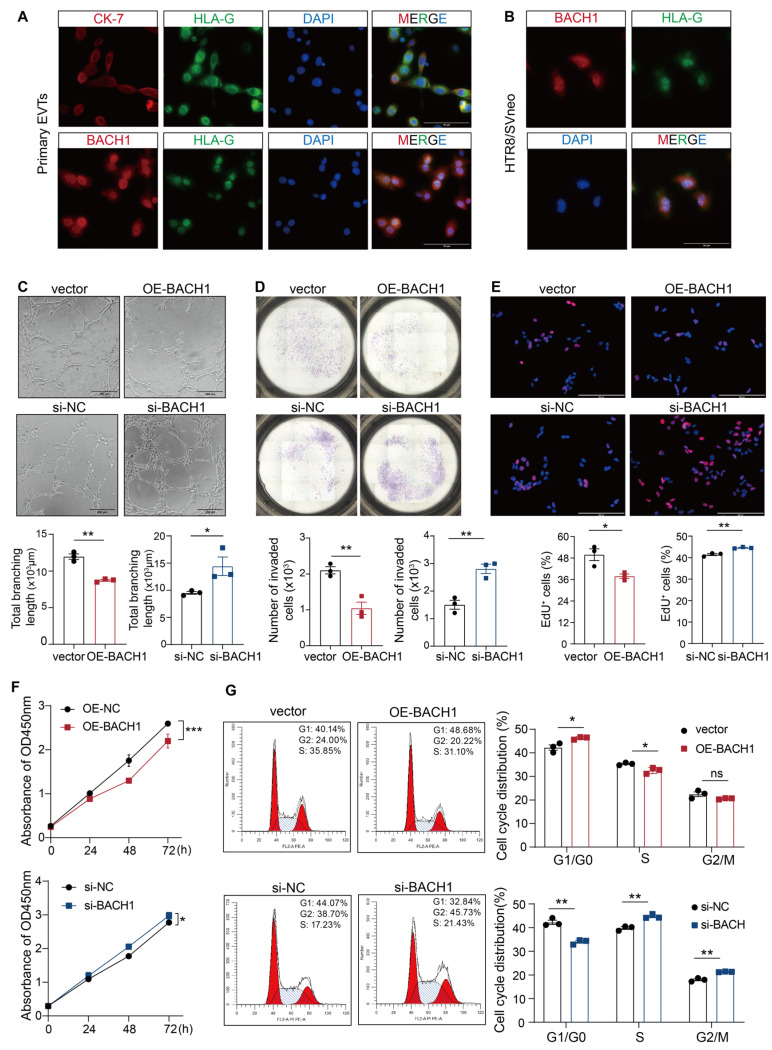
BACH1 regulates angiogenesis, invasion, proliferation, and cell cycle in HTR-8/SVneo Cells: (**A**) Immunofluorescence staining of primary extravillous trophoblasts (EVTs). Upper panel: Co-staining with the pan-trophoblast marker CK7 (red) and the EVT-specific marker HLA-G (green) to identify the cell population. Lower panel: Co-staining for BACH1 (red) and HLA-G (green) to demonstrate BACH1 expression and subcellular localization in EVTs. Nuclei are stained with DAPI (blue) in all images. Scale bar, 100 μm. (**B**) IF staining of HTR-8/SVneo cells showing co-localization of BACH1 (red) with the EVT marker HLA-G (green). Nuclei are stained with DAPI (blue). Scale bar, 100 μm. (**C**) Representative images (**upper**) and quantification (**lower**) of tube formation assays in HTR-8/SVneo cells with BACH1 overexpression or knockdown. (**D**) Representative images (**upper**) and quantification (**lower**) of Transwell invasion assays for HTR-8/SVneo cells with BACH1 overexpression or knockdown. (**E**) Representative images (**upper**) and quantification (**lower**) of 5-ethynyl-2’-deoxyuridine (EdU) incorporation assays in HTR-8/SVneo cells with BACH1 overexpression or knockdown. Nuclei are stained with Hoechst (blue). (**F**) Cell Counting Kit-8 (CCK-8) proliferation assays for HTR-8/SVneo cells with BACH1 overexpression or knockdown over 72 h. (**G**) Cell cycle analysis by propidium iodide (PI) staining and flow cytometry in HTR-8/SVneo cells with BACH1 overexpression or knockdown. Representative histograms (**left**) and quantification of cell cycle phase distribution (**right**) are shown. The horizontal axis (PE-A) represents propidium iodide (PI) fluorescence intensity, corresponding to cellular DNA content, and the vertical axis represents cell number. The first and second peaks correspond to the G0/G1 and G2/M phases, respectively, and the intervening region represents the S phase. The percentages of cells in each phase (G1, S, G2) are indicated. All quantitative data are presented as mean ± SEM from at least three independent experiments. Statistical significance was determined by unpaired two-tailed Student’s *t* test. * *p* < 0.05, ** *p* < 0.01, *** *p* < 0.001, ns, not significant.

Given the central role of EVT dysfunction in PE, we next investigated whether BACH1 directly regulates EVT biology. Immunofluorescence confirmed BACH1 expression in both the nucleus and cytoplasm of primary HLA-G+/CK7+ EVTs isolated from first-trimester villi (Figure 3A) and in the widely used EVT model HTR-8/SVneo cells (Figure 3B).

To determine the functional impact of BACH1 on EVTs, we first established gain-and loss-of-function models in HTR-8/SVneo cells via lentiviral overexpression or siRNA-mediated knockdown (Figure S2A–D). Modulating BACH1 levels bidirectionally altered key EVT functions. BACH1 overexpression attenuated angiogenic capacity (Figure 3C), impaired invasion (Figure 3D), and suppressed cell proliferation as measured by EdU incorporation and CCK-8 assays (Figure 3E,F). Conversely, BACH1 knockdown enhanced these functional parameters.

Mechanistically, flow cytometry revealed that BACH1 overexpression induced G0/G1 phase arrest, accompanied by a decrease in S-phase cells (Figure 3G). This was consistent with reduced protein levels of the cell cycle regulators CDK2 and cyclin A2 (Figure S2E,F). Notably, BACH1 did not significantly affect cell migration or apoptosis (Figure S2G,H), indicating a specific effect on proliferation-related pathways.

To further validate the inhibitory role of BACH1 across trophoblast lineages, we extended our observations to JEG-3 cells. Overexpression of BACH1 similarly suppressed invasion, tube formation, and proliferation in this distinct trophoblast model (Figure S3).

Collectively, these findings suggest that elevated BACH1 compromises trophoblast functions critical for placental development by promoting cell-cycle arrest and reducing proliferative, invasive, and angiogenic capacities. Such alterations in trophoblast function may contribute to impaired placental remodeling, a hallmark feature of PE.

### 3.4. BACH1 Overexpression Remodels the Metabolic Network in Trophoblasts

Given that cellular functions such as invasion and proliferation are highly energy-demanding, and considering the established role of BACH1 in repressing mitochondrial gene expression in other cell types [29], we hypothesized that it might impair EVT function by altering cellular metabolism. To test this, we performed untargeted metabolomic profiling on HTR-8/SVneo cells overexpressing BACH1 (OE-BACH1) versus vector controls.

**Figure 4:**
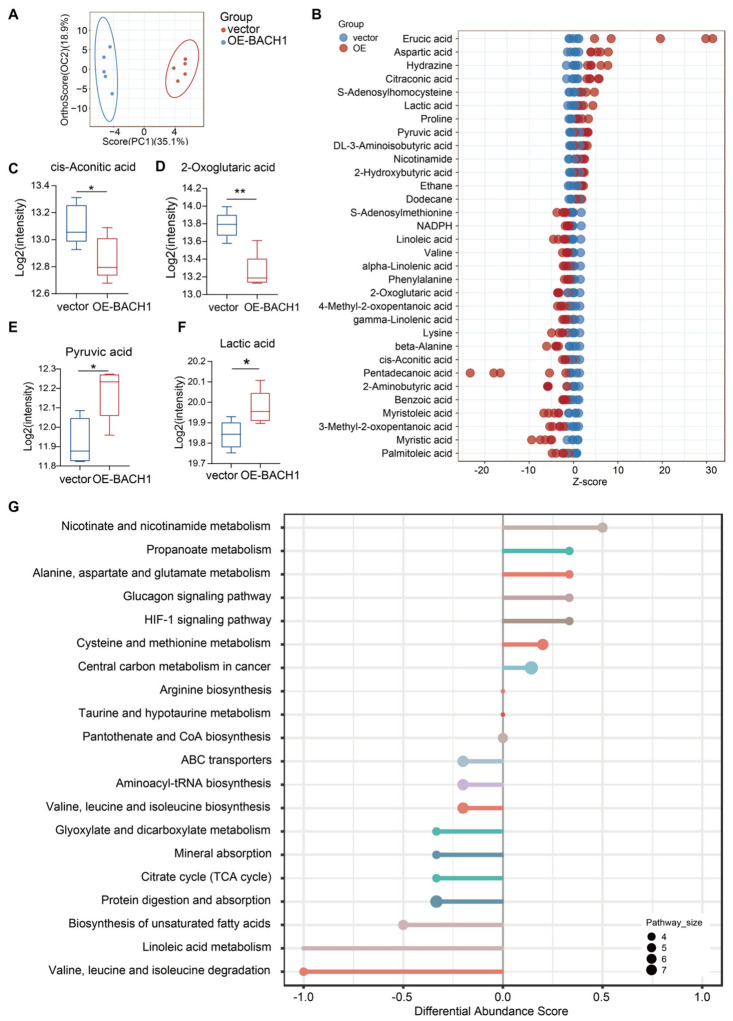
Metabolic alterations in HTR-8/SVneo Cells following BACH1 overexpression: (**A**) Orthogonal partial least squares-discriminant analysis (OPLS-DA) score plot derived from gas chromatography–mass spectrometry (GC–MS) metabolomics data, showing distinct metabolic profiles between control (vector) and BACH1-overexpressing (OE-BACH1) HTR-8/SVneo cells. (**B**) Z-score plot displaying the relative levels of 32 significantly differential metabolites (13 upregulated, 19 downregulated) identified between the two groups. (**C**–**F**) Quantitative analysis of selected differential metabolites: cis-aconitic acid (**C**), 2-oxoglutaric acid (**D**), pyruvic acid (**E**), and lactic acid (**F**). (**G**) Pathway analysis based on differential metabolite abundance. The bar plot shows the impact scores of significantly altered metabolic pathways, highlighting pronounced changes in mitochondrial-related processes including central carbon metabolism and the tricarboxylic acid (TCA) cycle upon BACH1 overexpression. Data are presented as mean ± SEM (**C**–**F**). Statistical significance was determined by unpaired two-tailed Student’s *t* test or the variable importance in projection (VIP) value > 1.0 and *p* < 0.05 in the OPLS-DA model. * *p* < 0.05, ** *p* < 0.01.

Orthogonal partial least squares discriminant analysis (OPLS-DA) revealed a distinct global metabolic shift induced by BACH1 (Figure 4A). We identified 32 significantly altered metabolites (13 upregulated, 19 downregulated; Figure 4B), with pronounced effects on mitochondrial pathways. Key intermediates of the TCA cycle, including cis-aconitate and 2-oxoglutarate, were significantly depleted (Figure 4C,D), indicating a bottleneck in central carbon metabolism. Concomitantly, the terminal glycolytic product lactate accumulated (Figure 4F), suggesting a compensatory shift toward glycolysis. Kyoto Encyclopedia of Genes and Genomes (KEGG) pathway analysis of the differential metabolites highlighted significant disturbances in “Central carbon metabolism” and the “TCA cycle” (Figure 4G), corroborating the targeted observations.

In summary, BACH1 overexpression substantially alters the trophoblast metabolic profile, characterized by reduced TCA cycle intermediates and increased glycolytic output. These metabolic alterations are consistent with a shift in cellular bioenergetic state and may contribute to the impaired trophoblast functions observed in BACH1-overexpressing cells.

### 3.5. BACH1 Compromises Mitochondrial Respiration and Enhances Glycolysis

**Figure 5:**
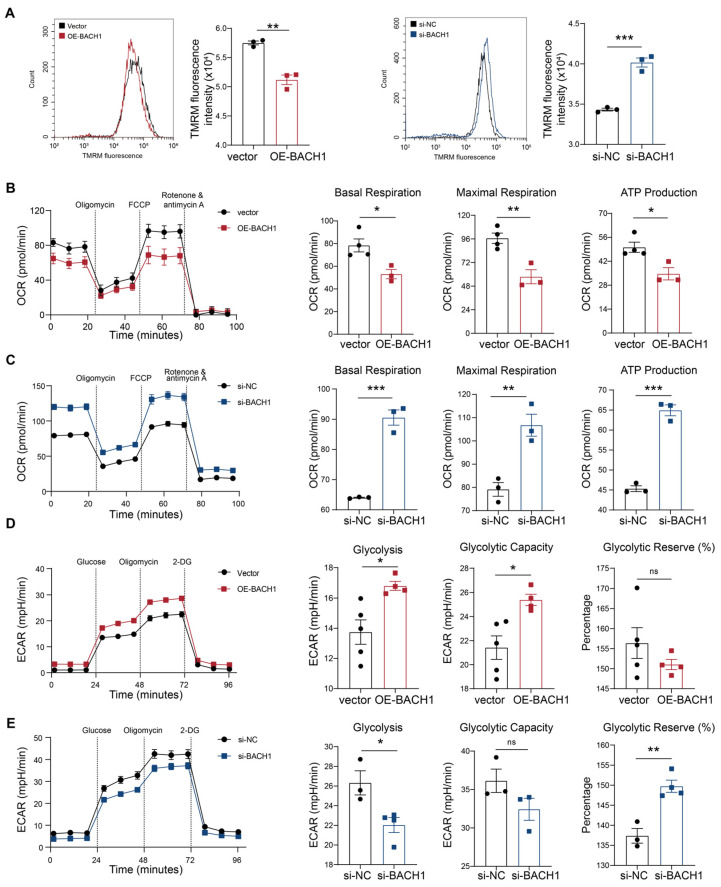
Impact of BACH1 on mitochondrial bioenergetics in HTR-8/SVneo Cells: (**A**) Representative images (**left**) and quantification (**right**) of tetramethylrhodamine methyl ester (TMRM) staining, indicating that BACH1 overexpression reduces mitochondrial membrane potential (ΔΨm), while knockdown increases it. (**B**,**C**) Mitochondrial stress test profiles (**left**) and quantification of key parameters (**right**) in HTR-8/SVneo cells with BACH1 overexpression (**B**) or knockdown (**C**). Parameters include basal respiration, maximal respiration, ATP production. (**D**,**E**) Glycolytic stress test profiles (**left**) and quantification of key parameters (**right**) in HTR-8/SVneo cells with BACH1 overexpression (**D**) or knockdown (**E**). Parameters include glycolysis, glycolytic capacity, and glycolytic reserve. Data are presented as mean ± SEM from at least three independent experiments. Statistical significance was determined by unpaired two-tailed Student’s *t* test. * *p* < 0.05, ** *p* < 0.01, *** *p* < 0.001; ns, not significant.

To define the bioenergetic consequences of the metabolic alterations induced by BACH1, we directly assessed mitochondrial function. BACH1 overexpression in HTR-8/SVneo cells increased reactive oxygen species (ROS) and reduced mitochondrial membrane potential (ΔΨm), while its knockdown had opposing effects (Figure 5A and Figure S4A,B), indicating a compromise in mitochondrial health.

We then utilized the Seahorse XFe24 Analyzer to perform real-time bioenergetic profiling. Mitochondrial stress tests revealed that BACH1 overexpression severely impaired OXPHOS, significantly decreasing basal respiration, maximal respiration, and ATP production (Figure 5B). Conversely, BACH1 knockdown enhanced these parameters (Figure 5C). Concurrently, glycolytic stress tests demonstrated that BACH1 overexpression increased both basal glycolysis and glycolytic capacity (Figure 5D). While BACH1 knockdown reduced basal glycolysis, it elevated the glycolytic reserve (Figure 5E), suggesting a dynamic adaptation to improved mitochondrial efficiency.

Together, these findings demonstrate that BACH1 overexpression substantially alters trophoblast bioenergetics, characterized by impaired mitochondrial respiration, reduced ATP-generating capacity, and increased reliance on glycolysis. The concurrent elevation of intracellular ROS and reduction in ΔΨm further support mitochondrial dysfunction as a key component of this metabolic shift.

### 3.6. BACH1 Overexpression Remodels the Mitochondrial Succinylome

Our data revealed that BACH1 overexpression suppresses OXPHOS and disrupts TCA cycle metabolite pools. Since Ksucc is a key mitochondrial post-translational modification known to directly regulate the activity of enzymes involved in these pathways, we postulated that BACH1 might induce metabolic dysfunction by remodeling the succinylome [37,38].

**Figure 6:**
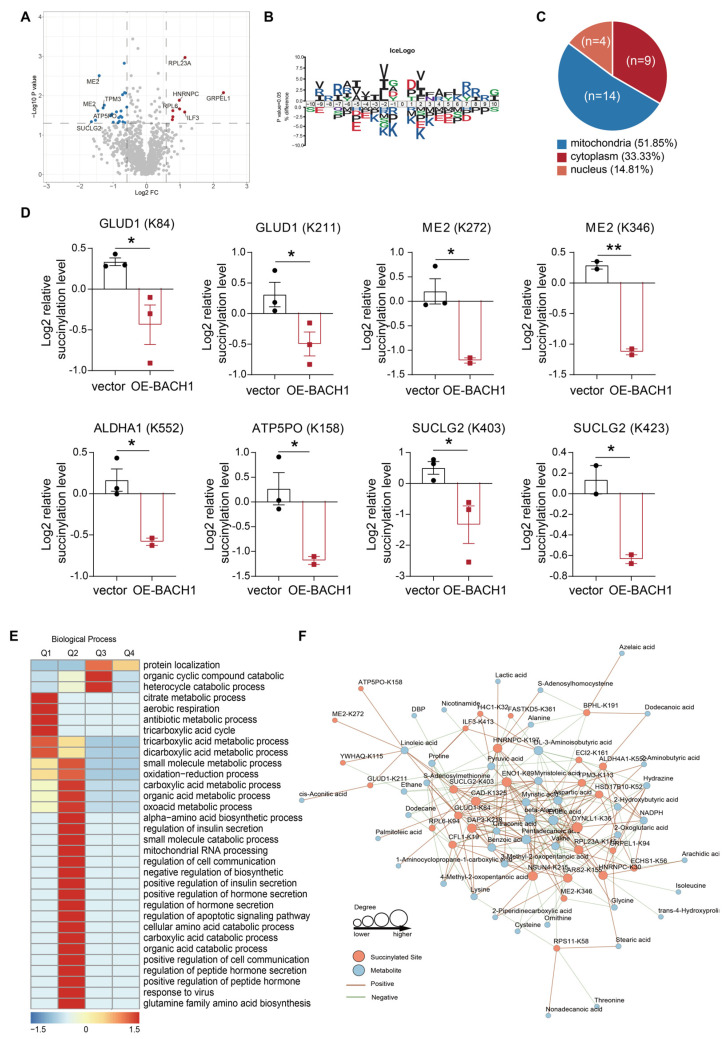
Succinylome profiling reveals altered mitochondrial protein succinylation following BACH1 overexpression: (**A**) Volcano plot displaying differentially lysine succinylation (Ksucc) sites in HTR-8/SVneo cells upon BACH1 overexpression. A total of 31 significant sites across 27 proteins were identified. Each dot represents a lysine succinylation site. Red dots indicate significantly upregulated sites and blue dots indicate significantly downregulated sites (fold change > 1.5 or <0.667, *p* < 0.05), while gray dots represent sites with no significant change. The dashed lines denote the fold-change and P-value thresholds. (**B**) Sequence motif analysis of the identified succinylation sites, visualized using iceLogo. The logo depicts the enrichment of specific amino acids flanking the succinylated lysine (center, denoted as “K”). Amino acids are colored according to their physicochemical properties (e.g., acidic, basic, polar, and hydrophobic residues), following the default iceLogo color scheme. Residues above the axis are enriched, and those below are depleted, at the indicated positions relative to the succinylated lysine (position 0); the vertical axis indicates the percentage difference, with significance set at *p* ≤ 0.05. (**C**) Subcellular localization analysis of the 27 differentially succinylated proteins. The pie chart illustrates that the majority are localized to mitochondria. (**D**) Analysis of differentially succinylated enzymes functionally annotated to “energy production and conversion” (based on COG/KOG database). (**E**) Functional enrichment analysis of differentially succinylated proteins stratified by fold change (Q1: <0.5; Q2: 0.5–0.667; Q3: 1.5–2; Q4: >2). (**F**) Integrated correlation network of Ksucc sites (orange) and metabolites (blue) (|ρ| > 0.8). Red and green edges denote positive and negative correlations, respectively. Node size corresponds to connectivity degree. Data are from a representative experiment of *n* = 3 independent biological replicates. * *p* < 0.05, ** *p* < 0.01.

To test this, we performed quantitative 4D succinylproteomics in control and BACH1-overexpressing HTR-8/SVneo cells. This revealed a significantly altered succinylome, with 31 differentially modified lysine sites across 27 proteins (Figure 6A). IceLogo analysis revealed a conserved sequence signature characterized by a significant enrichment of valine (V) at the −2 position and aspartic acid (D) at the +1 position flanking the succinylation sites. Notably, in the surrounding region (positions −3 to −1 and +1 to +3), lysine (K) residues were significantly depleted, suggesting a strong selective pressure against additional lysines in close proximity to the modification site (Figure 6B). Subcellular localization analysis showed that the majority of these targets were mitochondrial (Figure 6C), suggesting a close association between BACH1 overexpression and mitochondrial protein succinylation.

Notably, succinylation levels were altered at specific sites on several core metabolic enzymes, including GLUD1, ME2, ALDH4A1, ATP5PO, and SUCLG2 (Figure 6D). Functional enrichment analysis showed strong overrepresentation of these proteins in pathways related to citrate metabolism, aerobic respiration, and the TCA cycle (Figure 6E).

To integrate these findings with our metabolic data, we performed a correlation analysis between altered succinylation sites and differential metabolites. This revealed concerted enrichment in shared mitochondrial pathways, including the TCA cycle, pyruvate metabolism, OXPHOS, and glycolysis (Figure S5). Spearman correlation further mapped specific sites (e.g., GLUD1-K84, ME2-K346, SUCLG2-K403) to shifts in key metabolites (Figure 6F), suggesting a potential functional association.

In summary, BACH1 overexpression substantially alters the mitochondrial succinylome, affecting enzymes involved in central energy metabolism. These findings provide a mechanistic framework that may help explain the association between BACH1 overexpression and the observed bioenergetic alterations. Together, the data support the possibility that aberrant protein succinylation contributes to trophoblast dysfunction.

### 3.7. BACH1 Overexpression Is Associated with Elevated Protein Succinylation and Trophoblast Dysfunction

**Figure 7:**
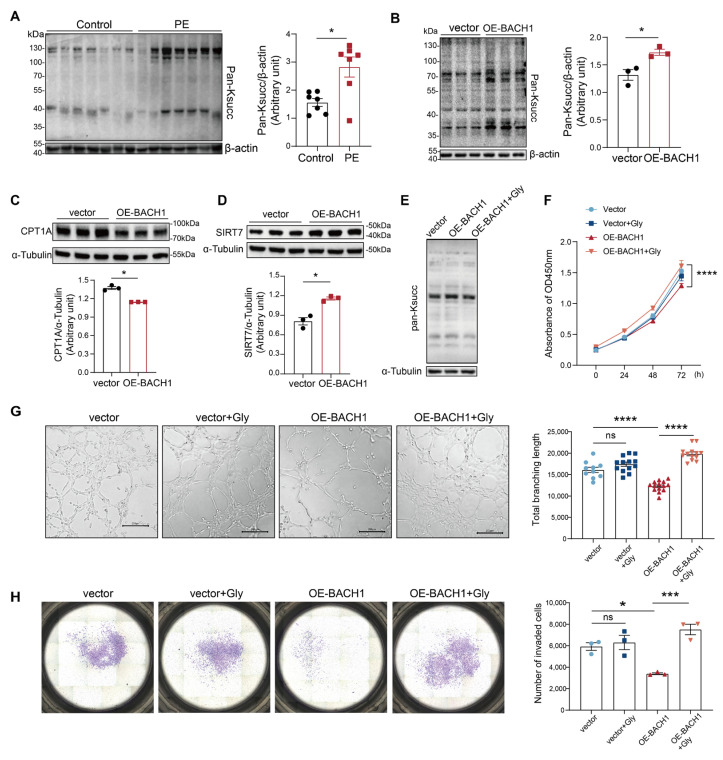
BACH1 elevates global protein succinylation and its functional rescue by glycine: (**A**) Western blot analysis of global lysine succinylation (pan-Ksucc) levels in placental tissues from PE patients and matched controls (*n* = 7 per group). β-actin served as the loading control. (**B**) Western blot analysis of pan-Ksucc levels in HTR-8/SVneo cells with BACH1 overexpression (OE-BACH1) compared to vector control. (**C**,**D**) Western blot analysis of the key succinyltransferase CPT1A (**C**) and the desuccinylase SIRT7 (**D**) in OE-BACH1 versus vector control HTR-8/SVneo cells. (**E**) Western blot analysis showing that treatment with glycine (10 mM) reduces the elevated global succinylation level induced by BACH1 overexpression in HTR-8/SVneo cells. (**F**–**H**) Functional rescue experiments in HTR-8/SVneo cells treated with glycine (10 mM). Glycine treatment restored the impaired proliferation ((**F**), CCK-8 assay), angiogenesis ((**G**), tube formation assay), and invasion ((**H**), Transwell assay) caused by BACH1 overexpression. Data are presented as mean ± SEM from at least three independent experiments. Statistical significance was determined by unpaired two-tailed Student’s *t* test (**A**–**D**) or one-way ANOVA (**E**–**H**). * *p* < 0.05, *** *p* < 0.001, **** *p* < 0.0001; ns, not significant.

To validate the functional relevance of the succinylome alterations, we first confirmed that global protein succinylation was significantly elevated in both PE placental tissues and BACH1-overexpressing HTR-8/SVneo cells (Figure 7A,B). We next explored whether the expression of enzymes involved in succinylation regulation was altered in association with the observed changes in global protein succinylation. Analysis of enzymes implicated in the regulation of protein succinylation revealed reduced CPT1A expression and increased SIRT7 expression in BACH1-overexpressing cells (Figure 7C,D). The potential relevance of these expression changes to PE was supported by independent data showing congruent expression changes for CPT1A [39] and SIRT7 in clinical PE samples (Figure S6D,E). No significant changes were observed for HAT1, KAT2A, or SIRT5 (Figure S6A–C).

Critically, we employed glycine as a pharmacological approach to attenuate protein succinylation. Glycine can be condensed with succinyl-CoA by δ-aminolevulinate synthase to generate 5-aminolevulinate, thereby channeling succinyl-CoA into the heme biosynthetic pathway and reducing the intracellular availability of succinyl-CoA for lysine succinylation. Previous studies have demonstrated that glycine supplementation effectively lowers succinyl-CoA levels, suppresses protein hypersuccinylation, and reverses succinylation-associated metabolic abnormalities in multiple cellular models [40,41]. Consistent with these observations, glycine treatment not only reduced the elevated succinylation in BACH1-overexpressing cells (Figure 7E) but also rescued the key functional defects: it restored proliferation (Figure 7F), tube formation capacity (Figure 7G), and invasive ability (Figure 7H).

Collectively, these findings indicate that BACH1 overexpression is associated with elevated global protein succinylation in trophoblasts. Altered expression of CPT1A and SIRT7 was observed in parallel with these succinylation changes, although the mechanistic relationship remains to be determined. Importantly, pharmacological reduction in protein succinylation partially rescued the proliferation, invasion, and angiogenic defects observed in BACH1-overexpressing cells. Together, these data support a functional contribution of aberrant protein succinylation to trophoblast dysfunction associated with BACH1 overexpression.

### 3.8. Trophoblast-Specific Bach1 Overexpression Recapitulates Key Features of PE in Mice

Having delineated the functional and metabolic consequences of BACH1 in vitro, we next investigated whether trophoblast-specific Bach1 overexpression could promote PE-like phenotypes in vivo. We employed a placental-targeted nanoparticle system conjugated to a placental chondroitin sulfate A-binding peptide (plCSA-BP), which delivers plasmids exclusively to trophoblasts [32].

**Figure 8:**
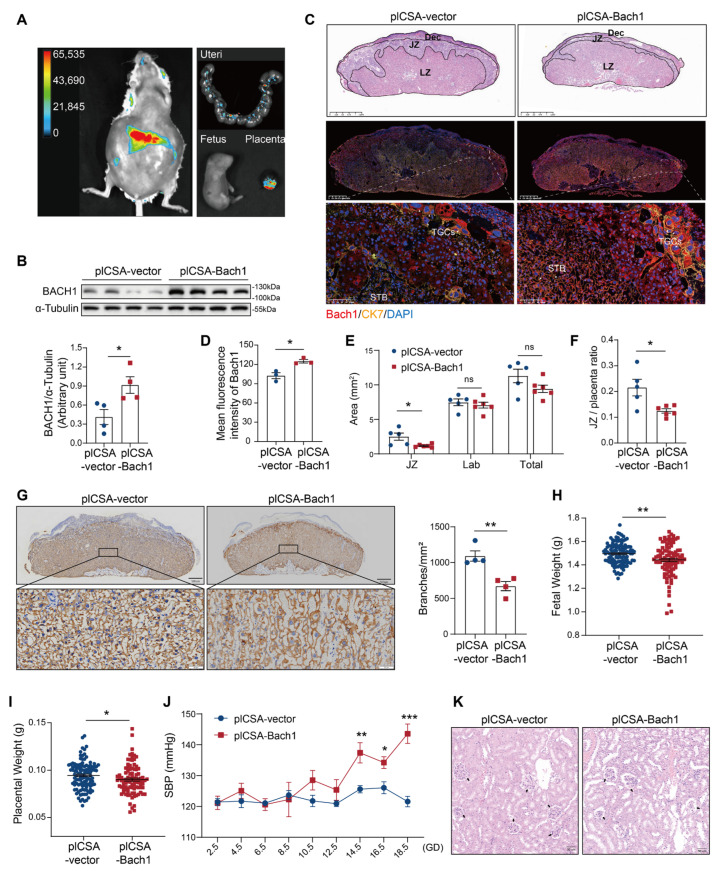
Trophoblast-specific Bach1 overexpression impairs placentation and induces PE-like phenotypes: (**A**) In vivo imaging 30 min post-intravenous placental chondroitin sulfate A-binding peptide (plCSA-BP) injection shows uterine fluorescence accumulation (**left**), with ex vivo imaging confirming placental-specific targeting (**right**). (**B**) Western blot analysis validating increased Bach1 protein expression in placental tissues from the plCSA-Bach1 group compared to the plCSA-vector control group at GD 18.5. (**C**) Representative H&E staining (**upper**) and IF staining (**lower**) of GD 18.5 placental sections. IF shows enhanced Bach1 signal (red) co-localizing with the CK7 (yellow), predominantly in the syncytiotrophoblast (STB) and trophoblast giant cells (TGCs). (**D**) Quantification of Bach1 immunofluorescence intensity confirms significant upregulation in the plCSA-Bach1 group. (**E**,**F**) Morphometric analysis of placental zones at GD 18.5 reveals a significantly reduced junctional zone (JZ) area in the plCSA-Bach1 group, with no significant change in the labyrinth zone (Lab) or total placental area. (**G**) Representative images of immunohistochemistry for CK7 in placental sections (upper panel: overview, scale bar = 500 µm; lower panel: magnified view, scale bar = 50 µm), demonstrating fetal vasculature branching. Quantification of branch points is shown on the right. (**H**,**I**) Fetal weight (**H**) and placental weight (**I**) at GD 18.5 are significantly reduced in the plCSA-Bach1 group. (**J**) Systolic blood pressure monitoring from GD 10.5 to 18.5 shows a significant elevation in the plCSA-Bach1 group from GD 14.5 onward. (**K**) Representative H&E-stained sections of maternal kidneys at GD 18.5 from the plCSA-vector and plCSA-Bach1 groups. Scale bar = 50 µm. Data are presented as mean ± SEM. Statistical significance was determined by unpaired two-tailed Student’s *t* test. * *p* < 0.05, ** *p* < 0.01, *** *p* < 0.001; ns, not significant.

Pregnant mice received intravenous injections of nanoparticles carrying a control (plCSA-vector) or a Bach1 overexpression plasmid (plCSA-Bach1) at key placentation timepoints (GD8.5, 10.5, 12.5). In vivo imaging confirmed specific placental accumulation of nanoparticles, with minimal signal in fetuses or non-placental tissues (Figure 8A). Western blot analysis validated robust Bach1 protein overexpression in placentas at GD18.5 (Figure 8B).

Immunofluorescence confirmed the trophoblast specificity of the manipulation, showing strong co-localization of Bach1 protein with CK7 and significantly increased signal intensity (Figure 8C,D). This targeted overexpression disrupted placentation, reducing both the junctional zone area (Figure 8E,F) and labyrinth vascular branching (Figure 8G).

Mice with trophoblast-specific Bach1 overexpression developed hallmark PE phenotypes: elevated systolic blood pressure from GD14.5 (Figure 8J), reduced placental and fetal weights at GD18.5 (Figure 8H,I), and glomerular constriction indicative of renal damage (Figure 8K).

Thus, trophoblast-specific Bach1 overexpression resulted in multiple PE-like features, including hypertension, fetal growth restriction, renal pathology, and impaired placental vascularization. These findings support a contributory role for trophoblast Bach1 upregulation in the development of PE-like phenotypes and are consistent with the hypothesis that elevated BACH1 participates in PE pathogenesis.

## 4. Discussion

The precise mechanisms initiating PE have not been fully elucidated. A widely accepted concept is that impaired remodeling of uterine spiral arteries leads to defective placentation, placental stress, and subsequent maternal syndrome. EVTs are central to this process and require coordinated mitochondrial metabolism to support proliferation, invasion, and vascular remodeling. BACH1 has been identified as an important regulator of mitochondrial metabolism [29], with potential effects on glycolysis, OXPHOS, the TCA cycle, and intracellular redox homeostasis. These observations raise the possibility that aberrant BACH1 activation may influence EVT metabolism and function. In the present study, we found that BACH1 was upregulated in PE placentas and early gestational maternal plasma from women who later developed PE. Experimental overexpression of BACH1 impaired trophoblast function, disrupted mitochondrial bioenergetics, and was accompanied by remodeling of lysine succinylation profiles in HTR-8/SVneo cells. Together, these findings support BACH1 as a PE-associated factor that can induce trophoblast metabolic dysfunction in experimental models and may contribute to the initiation and progression of PE.

Currently, limited evidence is available regarding the relationship between BACH1 and pregnancy-related complications, particularly PE. Our study revealed significant upregulation of BACH1 in both placental tissues at delivery and early pregnancy peripheral plasma from pregnancies that subsequently developed PE. The detection of increased BACH1 before clinical onset suggests that BACH1 elevation is unlikely to be merely a late consequence of established PE. This interpretation is further supported by our in vivo findings showing that BACH1 overexpression during the critical early placental developmental window was capable of inducing several PE-like phenotypes in pregnant mice. To consider potential influences of gestational age, we performed an additional analysis using the publicly available GSE75010 dataset. Placental BACH1 expression was not significantly associated with gestational age, indicating that the observed upregulation in PE placentas is unlikely to be driven primarily by gestational timing. This supports the notion that BACH1 elevation is a feature of PE rather than a reflection of normal developmental variation. Together, these observations suggest that excessive BACH1 activation may have a pathogenic role rather than representing only an adaptive response to placental stress.

Our in vivo experiments provide evidence that the aberrant upregulation of BACH1 in placental tissue can disrupt placentation and induce PE-like phenotypes in mice. In both the adenoviral system and the placenta-targeted delivery system, BACH1 overexpression reduced the junctional zone (JZ) area. The JZ, which contains spongiotrophoblast cells, glycogen cells, and trophoblast giant cells, plays an important endocrine and metabolic role in maintaining pregnancy [42,43]. Glycogen cells, located mainly in the JZ, serve as an energy source for the developing fetus, and numerous mouse models with JZ defects exhibit intrauterine growth restriction, associated with a decrease in the number of glycogen cells and a reduction in the total glycogen content [44]. Since PE and fetal growth restriction frequently coexist, the reduction in placental and fetal weights observed after BACH1 overexpression may be partly related to compromised JZ development and impaired placental metabolic support.

At the cellular level, BACH1 coordinately suppressed trophoblast angiogenic capacity, invasion, proliferation, and cell-cycle progression. Our findings align with previous investigations showing an inhibitory role of BACH1 in angiogenesis. In Bach1-deficient or low-expression mouse models, reduced BACH1 activity has been associated with improved recovery from hind-limb ischemia and increased vascular density [45], accompanied by enhanced angiogenic activity in vascular endothelial cells, including increased tube formation, migration, and proliferation, as well as elevated expression of Wnt/β-catenin downstream genes in HUVECs, such as IL-8 and VEGF; this inhibitory effect of BACH1 on angiogenesis is mediated primarily by the BTB domain [46]. Consistent with these findings, we found that BACH1 overexpression in HTR-8/SVneo cells significantly hindered tube formation and invasion, whereas BACH1 knockdown enhanced these functions. BACH1 also influenced trophoblast proliferation and cell-cycle progression. Consistent with its reported regulation of cell-cycle genes in other contexts, Warnatz et al. identified BACH1 target genes associated with cell-cycle control, including CDK6, MAFG, EWSR1, and LRRC8D, through chromatin immunoprecipitation sequencing and transcriptome profiling [47]. In our study, BACH1 overexpression induced G1/G0-phase cell cycle arrest and reduced the expression of cell-cycle regulators CDK2 and cyclin A2. Given the central role of impaired angiogenesis and defective trophoblast invasion in PE [48], and because placental development requires tight integration of proliferation, invasion, and angiogenesis, BACH1-induced disturbance of these EVT-like cell behaviors may represent one mechanism linking BACH1 elevation to PE-associated placental defects.

Metabolic remodeling is another major aspect of BACH1-associated trophoblast dysfunction. Lee et al. reported that BACH1 deletion or targeted degradation increases mitochondrial respiration in triple-negative breast cancer cells [29]. Specifically, BACH1 depletion increased basal and maximal OCR and decreased ECAR, while metabolomic analysis showed accumulation of TCA cycle intermediates and the downregulation of glycolytic intermediates. In contrast, we found that BACH1 overexpression weakened mitochondrial respiration, reduced ATP generation, and enhanced glycolytic activity in trophoblast cells, accompanied by decreased TCA cycle intermediates (such as cis-aconitic acid and α-ketoglutaric acid) and increased glycolytic metabolites (including pyruvic acid and lactate). These findings are directionally consistent with previous evidence that BACH1 suppresses mitochondrial oxidative metabolism and promotes glycolytic remodeling. The metabolic consequences of BACH1 may nonetheless vary across cell types, reflecting differences in basal metabolic state, biological function, epigenetic regulation, and microenvironmental context between trophoblast and tumor cells. In trophoblasts, a shift from mitochondrial oxidative metabolism toward glycolysis may reduce the bioenergetic reserve required for invasion, proliferation, and placental vascular remodeling. Consistently, our OCR/ECAR, ATP, ROS, ΔΨm, and metabolomic data support BACH1-associated bioenergetic disturbance in trophoblast cells.

An important aspect of our study is the association between BACH1 and Ksucc remodeling. Ksucc is a metabolically sensitive post-translational modification in which succinyl groups are covalently attached to lysine residues, thereby influencing protein charge, structure, and activity. Ksucc has been implicated in core energy metabolism pathways including the TCA cycle, glycolysis, and ketone metabolism [49,50]. Previous investigations have focused mainly on BACH1 as a negative transcriptional regulator that suppresses the expression of antioxidant and electron transport chain-related genes [29,51]. Our data suggest an additional layer of regulation, in which BACH1 overexpression is accompanied by altered Ksucc patterns in proteins involved in mitochondrial energy generation and conversion. In HTR-8/SVneo cells, BACH1 altered the abundance and/or succinylation status of several metabolism-associated proteins, including GLUD1, ME2, ALDH4A1, ATP5PO, and SUCLG2. Integrated analysis of succinylproteomics and metabolomics further showed that differentially succinylated proteins and metabolites were co-enriched in shared metabolic pathways, including OXPHOS, the TCA cycle, glycolysis, fatty acid synthesis, and amino acid degradation—pathways closely related to cellular energy production, biosynthesis, and mitochondrial homeostasis, the disruption of which may contribute to impaired trophoblast function. The regulatory connection itself appears complex: Ksucc is governed by both succinylation-related enzymes (such as CPT1A, KAT2A, and p300) and desuccinylases (including SIRT5 and SIRT7) [52], and the CPT1A and SIRT7 changes we observed indicate that BACH1 may influence components of this network. Although overall Ksucc was increased after BACH1 overexpression, the changes were protein- and site-specific, with several metabolic enzymes displaying reduced succinylation. SIRT7 upregulation may contribute to these site-specific desuccinylation events, while global hyper-succinylation may be driven by additional factors, including mitochondrial substrate availability, succinyl-CoA metabolism, CPT1A-related flux, mitochondrial stress.

The glycine rescue experiments further suggest that altered metabolism and Ksucc may participate in BACH1-induced trophoblast dysfunction. In HTR-8/SVneo cells, glycine treatment reduced BACH1-associated hyper-succinylation and partially restored proliferation, tube formation, and invasion, supporting the functional relevance of these alterations. Because glycine acts at the level of cellular metabolism—by reducing intracellular succinyl-CoA availability and thereby lowering global succinylation—rather than as a site-specific Ksucc inhibitor, its rescue effect is best interpreted as evidence for the involvement of a broader metabolic–succinylation axis rather than proof that individual succinylation events are functionally causal.

Several limitations should be considered when interpreting these findings. First, with respect to the clinical data, although our placental and plasma cohorts consistently showed elevated BACH1 in PE, the early-pregnancy plasma cohort was limited in size and was not designed to establish predictive performance. Larger prospective cohorts with independent validation, PE-subtype stratification, and multivariable adjustment will be required to define the relevance of BACH1 across different PE subtypes and to evaluate its potential value as a circulating indicator. Second, with respect to the experimental models, our two in vivo systems and complementary trophoblast cell lines are overexpression-based and therefore test whether elevated BACH1 is capable of disturbing placentation, but do not establish whether endogenous BACH1 is required for PE development; cell-line models also cannot fully recapitulate the physiological complexity of primary EVTs or the placental microenvironment. Future studies employing primary EVT cultures, trophoblast organoid systems, and trophoblast-specific Bach1 loss-of-function strategies will be necessary to define the requirement of endogenous BACH1 during PE progression. Third, with respect to mechanism, the association between BACH1 and Ksucc remodeling remains incompletely defined and largely correlative; direct functional validation—such as enzyme activity assays or lysine-to-arginine (K → R) mutagenesis—was not performed. Future studies using ChIP-seq, ChIP-qPCR, promoter-reporter assays, and site-specific functional analyses will be needed to clarify how BACH1 regulates this network and to identify the functionally critical succinylation sites.

## 5. Conclusions

In conclusion, our study identifies BACH1 as a PE-associated factor whose elevation precedes clinical manifestation and whose experimental overexpression impairs trophoblast function, placental development, and mitochondrial metabolic homeostasis. These findings support a potential contributory role for excessive BACH1 activation and suggest that mitochondrial Ksucc remodeling may contribute to trophoblast dysfunction. Together, the results establish a biologically plausible BACH1–mitochondrial metabolism–trophoblast dysfunction axis. Nevertheless, as these findings are based largely on overexpression models and association data, further validation—including loss-of-function studies, functional interrogation of succinylation targets, and larger prospective clinical cohorts—will be required to establish the causal role and clinical significance of BACH1 in PE.

## Figures and Tables

**Table 1:** Clinical characteristics of patients providing placental tissue.

Parameters	Control (*n* = 12)	PE (*n* = 12)	*p* Value
Age (years)	28.75 ± 2.05	29.33 ± 5.91	0.7499
BMI (kg/m^2^)	20.72 ± 2.89	21.81 ± 2.36	0.3223
Gestational age at diagnosis (weeks)	N/A	32.24 ± 1.36 *	N/A
Gestational age at delivery (weeks)	38.92 ± 1.82	35.75 ± 3.07	0.0055
Systolic Pressure (mmHg)	107.08 ± 10.73	163.00 ± 13.02	<0.0001
Diastolic Pressure (mmHg)	66.42 ± 8.96	102.92 ± 10.24	<0.0001
Neonatal Birth Weight (g)	3356.67 ± 524.96	2353.75 ± 826.72	0.0018
Urine Protein	neg to +	1+ to 4+	N/A

The data are presented as the means ± SDs and were analyzed via Student’s *t* test. BMI, body mass index. * Gestational age at diagnosis was applicable only to the PE group and was therefore not included in between-group statistical comparisons.

**Table 2:** Clinical characteristics of patients who provided peripheral blood.

Parameters	Control (*n* = 10)	PE (*n* = 10)	*p* Value
Age (years)	31.10 ± 2.56	32.40 ± 4.09	0.4052
BMI (kg/m^2^)	20.55 ± 2.68	22.28 ± 2.39	0.1465
Gestational age at blood collection (weeks)	12.86 ± 1.01	13.51 ± 1.30	0.2226
Gestational age at diagnosis (weeks)	N/A	32.21 ± 1.91 *	N/A
Gestational age at delivery (weeks)	39.89 ± 0.91	34.49 ± 3.57	0.0002
Systolic Pressure (mmHg)	115.30 ± 14.02	166.60 ± 19.81	<0.0001
Diastolic Pressure (mmHg)	68.30 ± 8.93	103.30 ± 13.03	<0.0001
Neonatal Birth Weight (g)	3472.00 ± 313.77	2094.50 ± 841.15	0.0001
Urine Protein	neg to +	1+ to 4+	N/A

The data are presented as the means ± SDs and were analyzed via Student’s *t* test. BMI, body mass index. * Gestational age at diagnosis was only applicable to the PE group and therefore was not included in between-group statistical comparisons.

**Table 3:** Primer sequences used for qRT-PCR analysis.

Gene	Direction	Sequence (5′–3′)
BACH1	Forward	CGCCTCAGCTCTGGTTGAT
	Reverse	TTCCGCTGGTCATTAAGGCT
β-actin	Forward	TGGCACCCAGCACAATGAA
	Reverse	CTAAGTCATAGTCCGCCTAGAAGCA

Relative expression levels were determined through the 2^−△△CT^ method.

## Data Availability

The mass spectrometry datasets generated in this study have been deposited in the OMIX, China National Center for Bioinformation/Beijing Institute of Genomics, Chinese Academy of Sciences [53,54] (accession numbers OMIX015392 for metabolomics and OMIX015360 for succinyllysine proteomics). They are publicly accessible through the OMIX repository (https://ngdc.cncb.ac.cn/omix, accessed on 3 March 2026). Additionally, the datasets used and/or analyzed during the current study are available from the corresponding author upon reasonable request.

## Supplementary Materials

The following supporting information can be downloaded at: https://www.mdpi.com/article/10.3390/antiox15070835/s1, Supplementary Material S1: Supplementary methods, Table S1 (Primary antibodies used for Western blot analysis), and Figures S1–S6, including validation of placental transduction efficiency, BACH1 modulation efficiency, additional functional analyses in trophoblast cells, ROS detection, integrated KEGG pathway enrichment analysis, and regulation of succinylation-related enzymes. Supplementary Material S2: Full uncropped scans of all Western blots used in the main manuscript and supplementary figures.

## Abbreviations

PE	Preeclampsia
EVTs	Extravillous Trophoblasts
OXPHOS	Oxidative Phosphorylation
Ksucc	Lysine Succinylation
TCA cycle	Tricarboxylic Acid Cycle
BACH1	BTB and CNC Homology 1
GEO	Gene Expression Omnibus
ACOG	American College of Obstetricians and Gynecologists
ELISA	Enzyme-Linked Immunosorbent Assay
CV	Coefficient of Variation
qRT–PCR	Quantitative Real-Time PCR
SDS-PAGE	Sodium Dodecyl Sulfate–Polyacrylamide Gel Electrophoresis
PVDF	Polyvinylidene Difluoride
HRP	Horseradish Peroxidase
ECL	Enhanced Chemiluminescence
GD	Gestational Day
ICR	Institute of Cancer Research
PFU	Plaque-Forming Units
NPs	Nanoparticles
PLGA	Poly(lactic-co-glycolide)
plCSA-BP	Placental Chondroitin Sulfate A-Binding Peptide
PDI	Polydispersity Index
ICG	Indocyanine Green
H&E	Hematoxylin and Eosin
IHC	Immunohistochemistry
FBS	Fetal Bovine Serum
EMEM	Eagle’s Minimum Essential Medium
MOI	Multiplicity of Infection
EdU	5-Ethynyl-2′-deoxyuridine
CCK-8	Cell Counting Kit-8
GC-MS	Gas Chromatography–Mass Spectrometry
TMRM	Tetramethylrhodamine Methyl Ester
ΔΨm	Mitochondrial Membrane Potential
MFI	Mean Fluorescence Intensity
OCR	Oxygen Consumption Rate
ECAR	Extracellular Acidification Rate
SEM	Standard Error of the Mean
OPLS-DA	Orthogonal Partial Least Squares Discriminant Analysis
KEGG	Kyoto Encyclopedia of Genes and Genomes
ROS	Reactive Oxygen Species
SpT	Spongiotrophoblast
GC	cells Glycogen Cells
TGC	Trophoblast Giant Cells
TNBC	Triple-Negative Breast Cancer
ETC	Electron Transport Chain
JZ	Junctional Zone
