# Supplementary material for: Elevated BACH1 Contributes to Mitochondrial Succinylome Remodeling and Trophoblast Bioenergetic Dysfunction in Preeclampsia
Source: Antioxidants (Basel). 2026 Jul 1;15(7):835. doi: 10.3390/antiox15070835 (PMC13403510; doi:10.3390/antiox15070835)
Supplement: Supplementary file 1 [file antioxidants-15-00835-s001.zip › Supplementary Material S1.pdf]

Supplementary Materials

Table S1：Primary antibodies used for Western blot analysis.

Target	Host	Catalog Number	Manufacturer	Dilution
BACH1	Rabbit	14018-1-AP	Proteintech	1:1000
CDK2	Mouse	60312-1-Ig	Proteintech	1:1000
Cyclin A2	Mouse	66391-1-Ig	Proteintech	1:2000
α-Tubulin	Mouse	66031-1-Ig	Proteintech	1:20000
β-actin	Mouse	66009-1-Ig	Proteintech	1:20000
GAPDH	Mouse	60004-1-Ig	Proteintech	1:20000
anti-succinyllysine	Mouse	PTM-419	PTM BIO	1:1000
CPT1A	Rabbit	15184-1-AP	Proteintech	1:5000
KAT2A	Rabbit	3305T	Cell Signaling Technology	1:1000
HAT1	Rabbit	ab194296	Abcam	1:1000
SIRT5	Rabbit	ab259967	Abcam	1:1000
SIRT7	Rabbit	5360	Cell Signaling Technology	1:1000

Supplementary Methods

Wound-healing Assay

The migration ability of post-treated HTR8/SVneo cells was assessed using a wound-healing assay. Cells were cultured in a 6-well plate until reaching over 90% confluence. A straight line was drawn through the cells, and after washing with PBS, the medium was replaced with fresh serum-free culture medium. The plate was then placed in a 37℃, 5% CO_2 incubator for 24 hours. Images were captured using the EVOS FL Color Imaging System, and the scratch closure area was quantified with ImageJ 1.53o

software.

Apoptosis Detection

Apoptosis in HTR8/SVneo cells was detected using the Annexin V-FITC/PI Apoptosis Kit (Elabscience, China) and Annexin V-APC/PI Apoptosis Kit (Elabscience, China) combined with flow cytometry. Equal numbers of HTR8/SVneo cells from each group were seeded in a 6-well plate and incubated in a 37°C CO_2 incubator. Cells were washed with PBS and digested using trypsin. A total of 1×10^6 cells were resuspended in 500 µl of PBS without Ca^{2+} and Mg^{2+}. Subsequently, 500 µl of diluted 1× Annexin V Binding Buffer was added and gently mixed. For the vector and OE-BACH1 groups, 5 µl of Annexin V-APC Reagent and 5 µl of PI Reagent (50 µg/ml) were added. For the si-NC and si-BACH1 groups, 5 µl of Annexin V-FITC Reagent and 5 µl of PI Reagent (50 µg/ml) were added. After gentle mixing, cells were incubated in the dark at room temperature for 15 minutes. The stained cell suspensions were then analyzed using a flow cytometer, and data analysis was performed using the CytExpert_2.4 software.

Plasmid Transfection in JEG-3 Cells

The full-length human BACH1 overexpression plasmid (pCMV3-BACH1) and corresponding empty vector control (pCMV3-ctrl) were commercially sourced from Hanheng Biotechnology Co., Ltd. (Shanghai, China). For transfection, JEG-3 cells were seeded in 6-well plates at a density of 2×10^5 cells/well and cultured overnight in EMEM supplemented with 10% FBS. Transfection was initiated when cells reached 60–80% confluence. Using Lipofectamine™ 3000 reagent (Invitrogen) according to the manufacturer's protocol, 1 µg of plasmid DNA (BACH1 or empty vector) was complexed with 2 µL of P3000™ enhancer reagent in Opti-MEM™ Reduced-Serum Medium (Gibco). The DNA-lipid complexes were added dropwise to cells and incubated for 6 h at 37°C/5% CO_2, followed by replacement with complete growth medium. Cells were harvested at 24 h or 48 h post-transfection for subsequent analyses.

ROS Detection

ROS levels in HTR8/SVneo cells were measured using the superoxide anion fluorescence probe dihydroethidium (DHE) (Beyotime, China) and analyzed with the CytoFLEX Flow Cytometer. Cells were incubated with serum-free culture medium

containing 2 μM DHE at 37°C for 30 minutes to load the fluorescence probe, followed by washing. For ROS detection using DCFH-DA, a Reactive Oxygen Species Assay Kit (Beyotime, China) was employed. DCFH-DA (10 μM) was added, and after incubation and washing, fluorescence was detected using a microplate reader (Tecan Spark, Switzerland).

Supplementary Figures

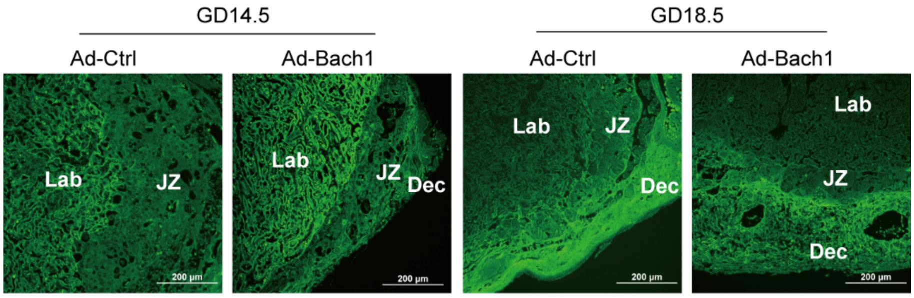

Figure S1. Validation of targeted transduction efficiency in mouse placental tissue Representative immunofluorescence images detecting Enhanced Green Fluorescent Protein (EGFP) expression on frozen sections of placental tissue from pregnant mice at GD 14.5 and GD 18.5. EGFP serves as a reporter for the successful transduction of the targeted delivery system. Images demonstrate specific and efficient expression within the placental labyrinth and junctional zones at both time points (n = 3 placentas per group). Scale bar = 200 μm.

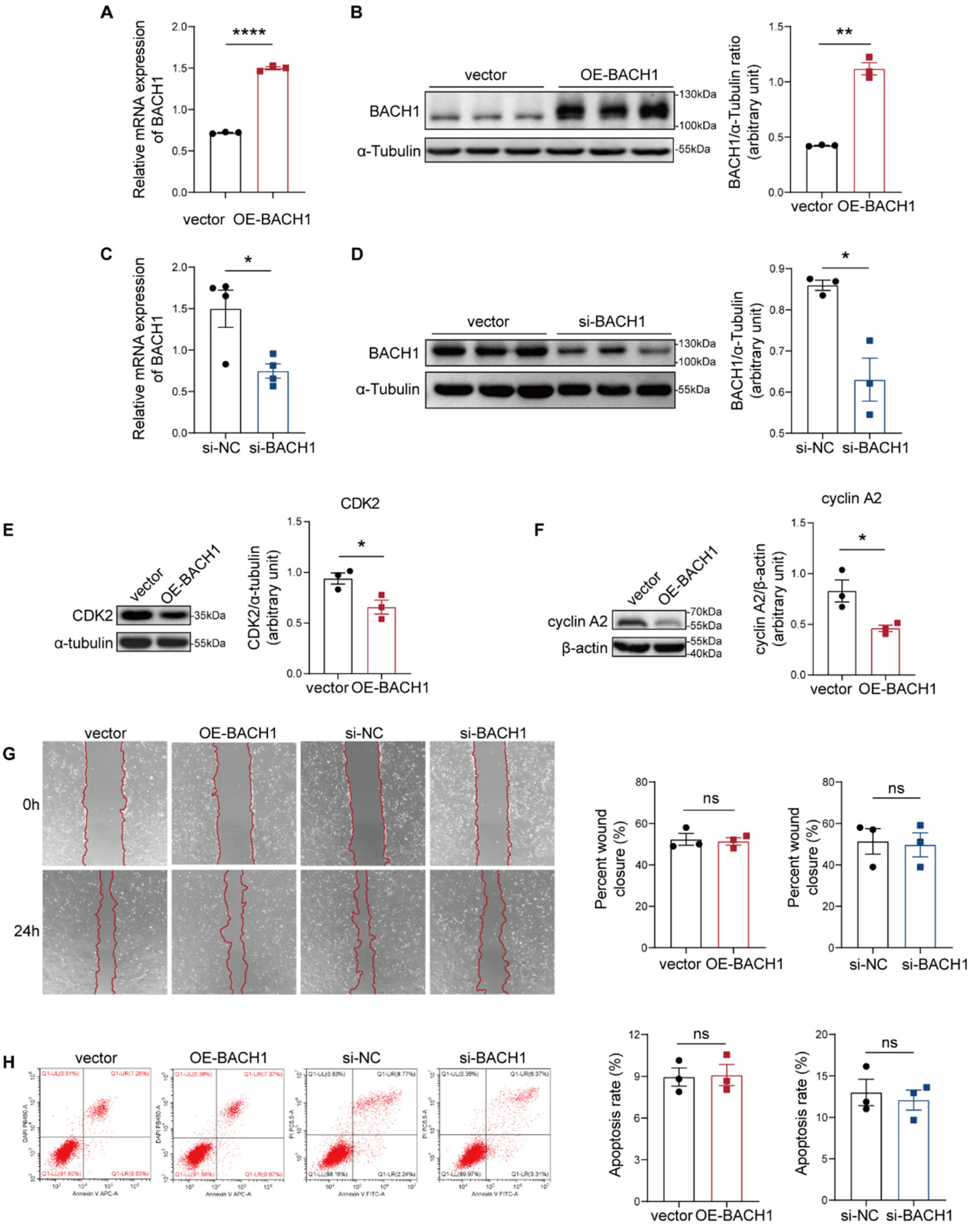

Figure S2. Validation of BACH1 modulation and assessment of its functional effects in HTR8/SVneo cells

(A, B) Validation of BACH1 overexpression. qRT-PCR (A) and representative western blot (B) analyses of BACH1 expression in HTR8/SVneo cells transduced with lentivirus carrying BACH1 (OE) or empty vector control (Vector). (C, D) Validation of BACH1 knockdown. qRT-PCR (C) and representative western blot (D) analyses of BACH1 expression in HTR8/SVneo cells transfected with BACH1-specific siRNA

(siBACH1) or non-targeting scrambled siRNA (si-NC). (E, F) Western blot analysis of the cell cycle regulators CDK2 (E) and cyclin A2 (F) following BACH1 overexpression. (G) Cell migration capacity assessed by wound healing assay. Representative images (left) and quantification of wound closure area (right) at 24 hours are shown. (H) Apoptosis analysis by flow cytometry using Annexin V and propidium iodide (PI) staining. The bar graph shows the percentage of apoptotic cells (early + late apoptosis). Data are presented as mean ± SEM from at least three independent experiments. Statistical significance was determined by unpaired two-tailed Student's t test. *P < 0.05, **P < 0.01, ****P < 0.0001; ns, not significant.

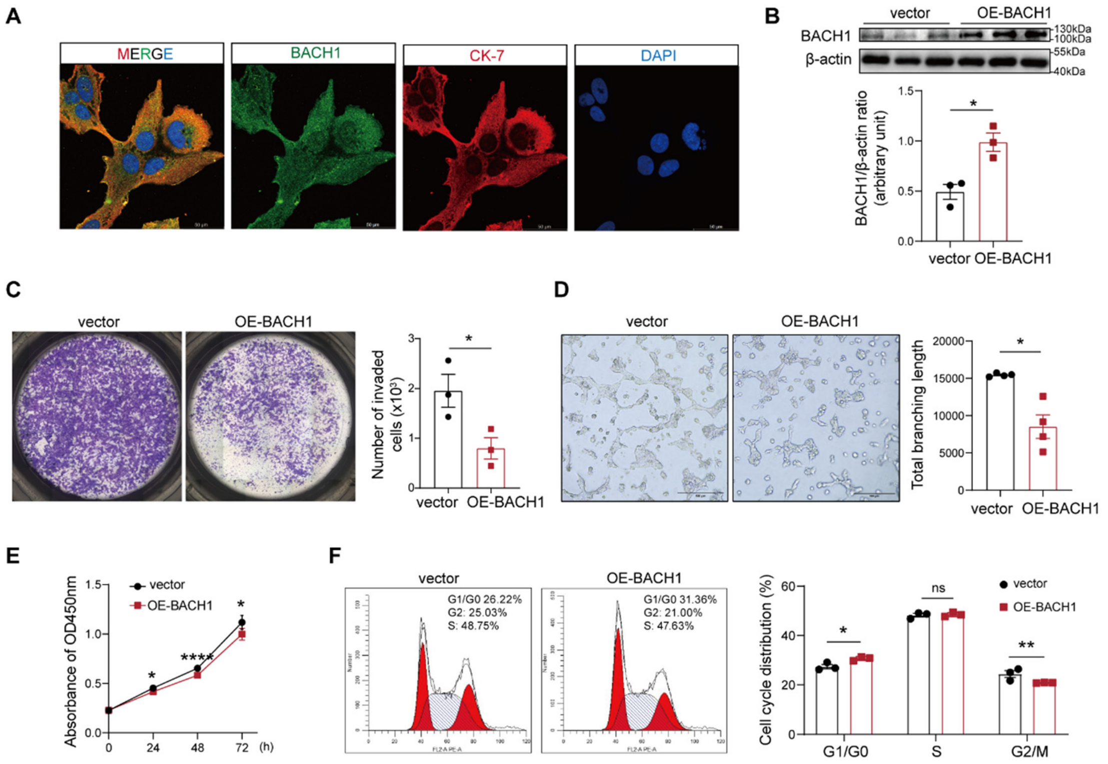

Figure S3. Overexpression of BACH1 inhibits invasion, angiogenesis, and proliferation in JEG-3 trophoblast cells

(A) IF staining of BACH1 (green) co-localizing with the trophoblast marker CK7 (red) in JEG-3 cells. Nuclei are stained with DAPI (blue). Scale bar, 50 µm. (B) Western blot analysis validating BACH1 protein overexpression in JEG-3 cells transfected with a BACH1 expression plasmid (OE) compared to the empty vector control (Vector). β-actin served as the loading control. (C) Transwell invasion assay.

Representative images (left) and quantification (right) show that BACH1 overexpression significantly reduces the invasive capacity of JEG-3 cells. (D) Tube formation assay. Representative images (left) and quantification (right) demonstrate impaired angiogenic ability in BACH1-overexpressing JEG-3 cells. (E) Cell proliferation assessed by CCK-8 assay. BACH1 overexpression reduces the proliferation of JEG-3 cells over 72 hours. (F) Cell cycle analysis by flow cytometry. Representative histograms (left) and quantification (right) indicate that BACH1 overexpression induces G1/G0 phase arrest in JEG-3 cells. Data are presented as mean ± SEM from at least three independent experiments. Statistical significance was determined by unpaired two-tailed Student's t test. *$P < 0.05$, **$P < 0.01$, ****$P < 0.0001$; ns, not significant.

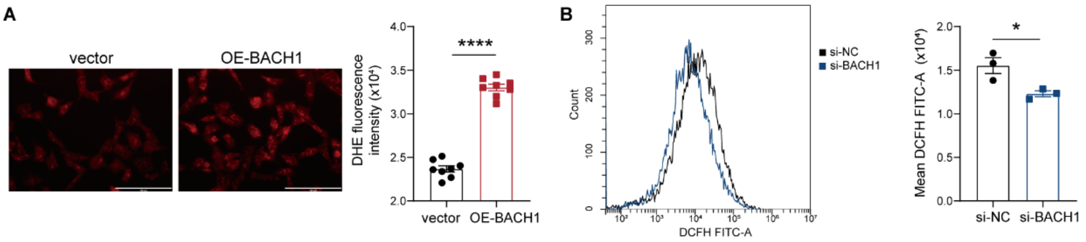

Figure S4. BACH1 modulates reactive oxygen species (ROS) generation in HTR8/SVneo trophoblast cells

(A) Measurement of intracellular ROS levels using dihydroethidium (DHE) fluorescence. Representative fluorescence images (left) and quantification of mean fluorescence intensity (right) show that BACH1 overexpression significantly increases ROS levels in HTR8/SVneo cells. (B)Analysis of intracellular ROS production using the fluorescent probe 2',7'-dichlorodihydrofluorescein diacetate (DCFH-DA) by flow cytometry. Representative histogram overlays (left) and quantification of the mean fluorescence intensity (right) demonstrate that BACH1 knockdown attenuates ROS production. Data are presented as mean ± SEM from at least three independent experiments. Statistical significance was determined by unpaired two-tailed Student's t test, comparing BACH1-modulated groups to their respective controls (vector or scrambled siRNA). *$P < 0.05$, ****$P < 0.0001$.

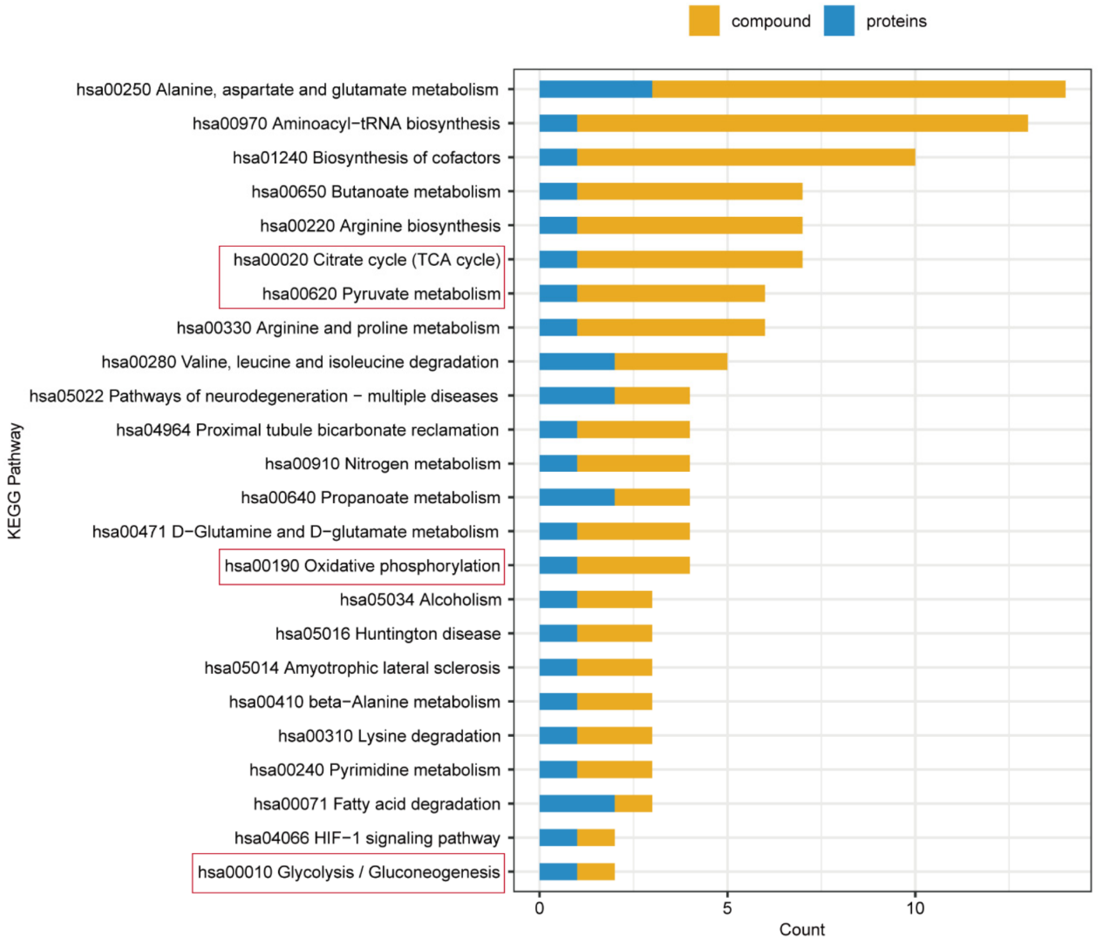

Figure S5. Integrated KEGG pathway enrichment analysis of the succinylome and metabolome

The bar chart displays the combined KEGG pathway enrichment results for differentially succinylated proteins and altered metabolites identified in BACH1-overexpressing HTR8/SVneo cells. Pathways are ranked by their combined enrichment significance. Key significantly enriched pathways include Alanine, aspartate and glutamate metabolism, Citrate cycle (TCA cycle), Pyruvate metabolism, and Oxidative phosphorylation, highlighting a profound impact of BACH1 on central mitochondrial metabolic and amino acid pathways. Enrichment significance was calculated from the corresponding omics datasets (n = 3). Pathways with a combined enrichment score > 2.0 and $P < 0.05$ are shown.

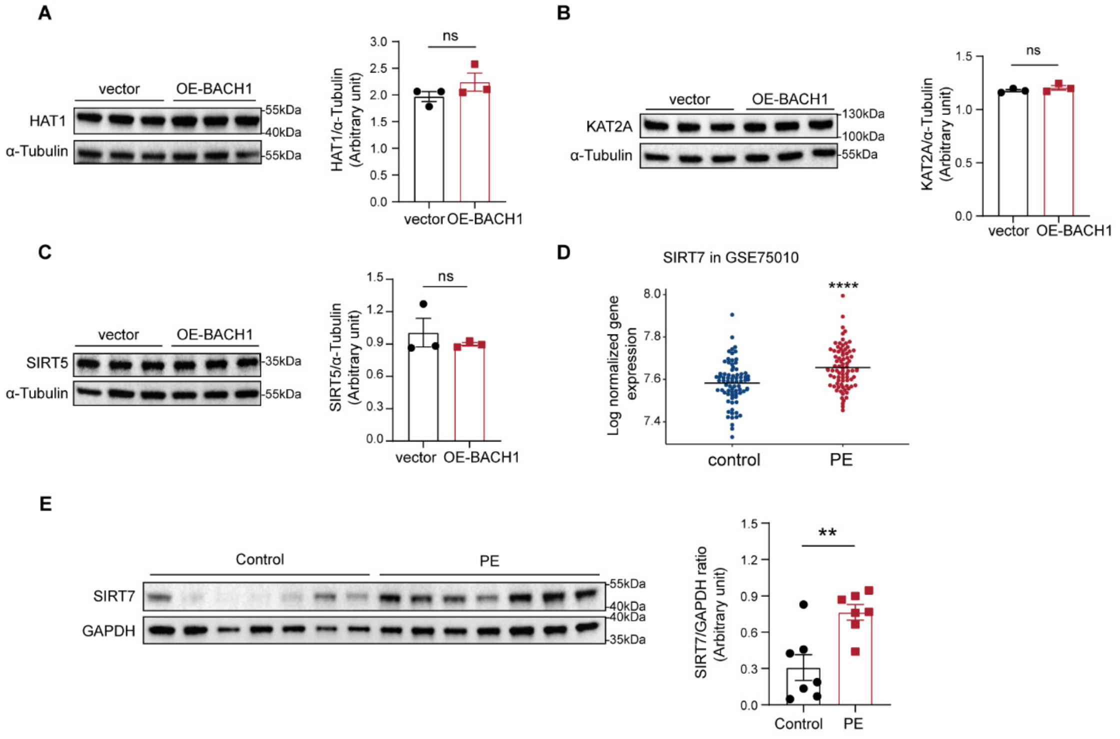

Figure S6. BACH1 regulates succinylation enzymes and validates SIRT7 association with preeclampsia

(A–C) Western blot analysis of key lysine succinylation-modifying enzymes in HTR8/SVneo cells with BACH1 overexpression (OE-BACH1) versus vector control. Expression levels of the succinyltransferases HAT1 (A) and KAT2A (B), and the desuccinylase SIRT5 (C) are shown. α-Tubulin served as the loading control. (D) SIRT7 mRNA expression in the GSE75010 placental transcriptome dataset (Control, n = 77; PE, n = 80). (E) Representative western blot (left) and quantification (right) of SIRT7 protein levels in human term placental tissues (Control, n = 7; PE, n = 7). GAPDH served as the loading control. Data are presented as mean ± SEM from at least three independent experiments. Statistical significance was determined by unpaired two-tailed Student's t test. $^{**}P < 0.01$, $^{****}P < 0.0001$; ns, not significant.
