# Supplementary material for: Elevated BACH1 Contributes to Mitochondrial Succinylome Remodeling and Trophoblast Bioenergetic Dysfunction in Preeclampsia
Source: Antioxidants (Basel). 2026 Jul 1;15(7):835. doi: 10.3390/antiox15070835 (PMC13403510; doi:10.3390/antiox15070835)
Supplement: Supplementary file 1 [file antioxidants-15-00835-s001.zip › Supplementary Material S2.pdf]

Full Uncropped Scans of Western Blots

This file contains the original, uncropped scans of all western blots used to generate the key figures in the main manuscript and supplementary materials.

1. Figure 1J: BACH1 in human placenta (Control n=7, PE n=7).

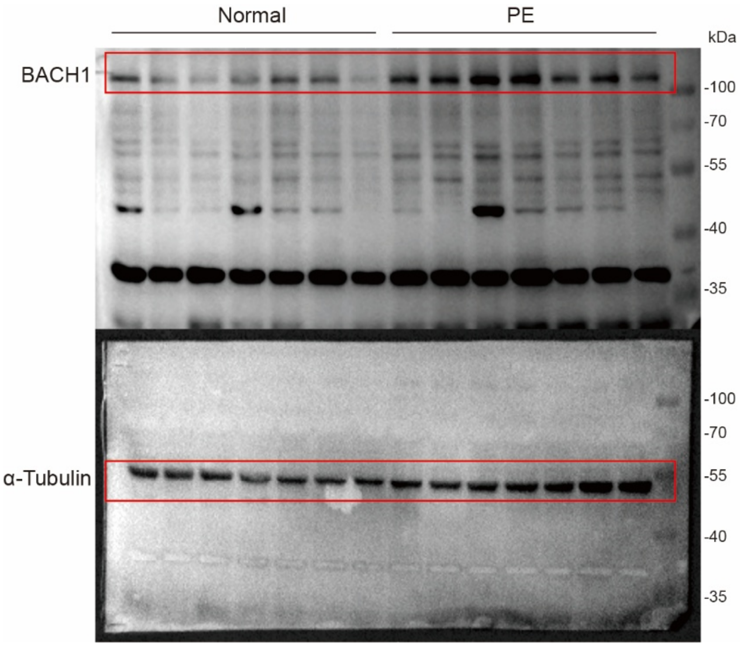

2. Figure 2B: Bach1 in mouse placenta at GD14.5 (Ad-Ctrl n=3, Ad-Bach1 n=3).

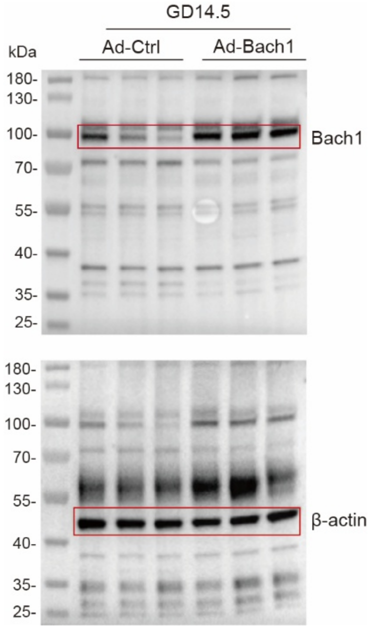

3. Figure 2C: Bach1 in mouse placenta at GD18.5 (Ad-Ctrl n=5, Ad-Bach1 n=5).

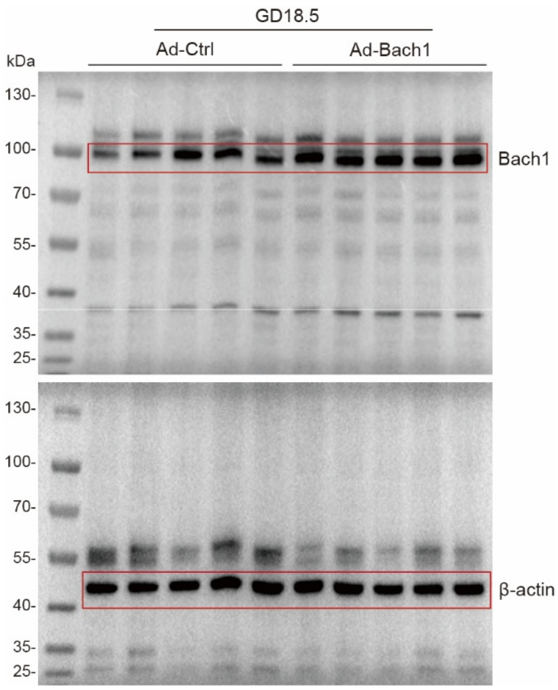

4. Figure 7A: Protein succinylation (Ksucc) in human placenta (Control n=7, PE n=7).

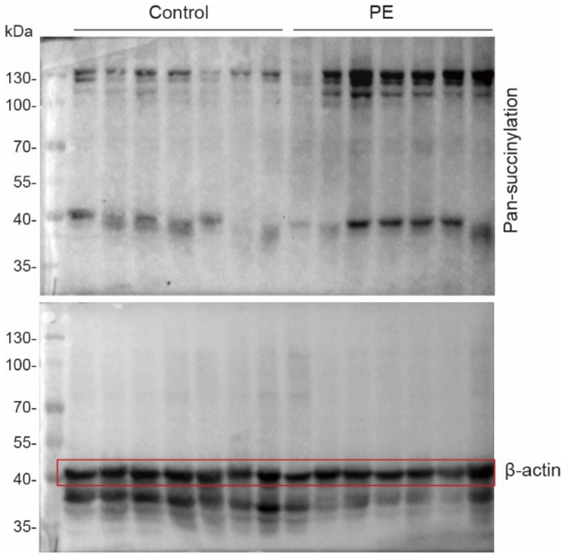

5. Figure 7B: Protein succinylation (Ksucc) in HTR8/SVneo cells (Vector n=3, OE-BACH1 n=3).

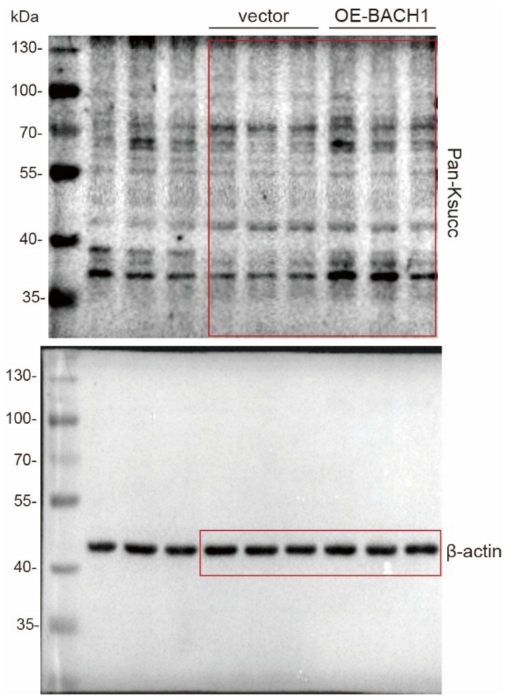

6. Figure 7C: CPT1A in HTR8/SVneo cells (Vector n=3, OE-BACH1 n=3).

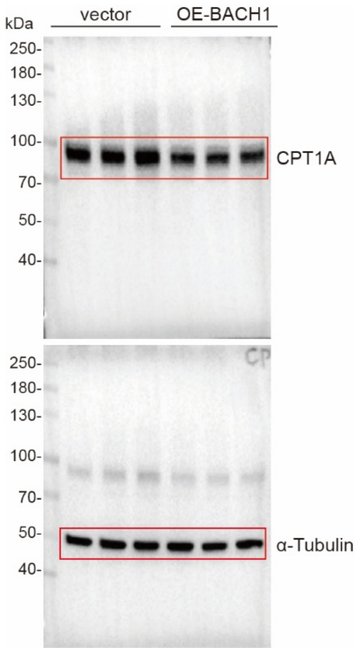

7. Figure 7D: SIRT7 in HTR8/SVneo cells (Vector n=3, OE-BACH1 n=3).

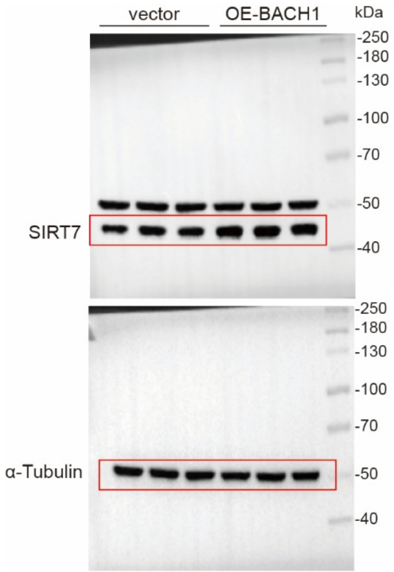

8. Figure 7E: Effect of Glycine on Ksucc in OE-BACH1 HTR8/SVneo cells.

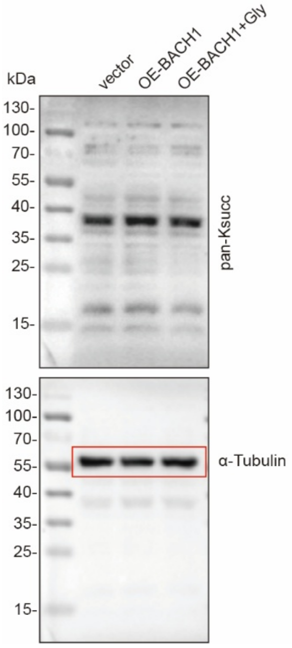

9. Figure 8B: Bach1 in targeted-nanoparticle model placenta (plCSA-vector n=4, plCSA-Bach1 n=4).

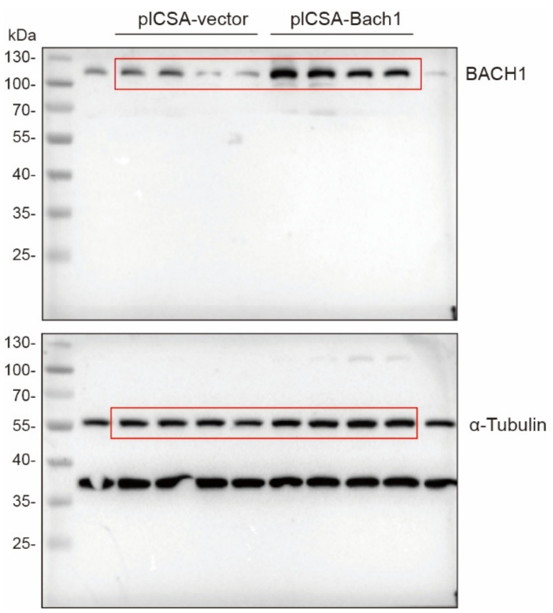

10. Figure S2B: BACH1 overexpression in HTR8/SVneo cells (n=3 per group).

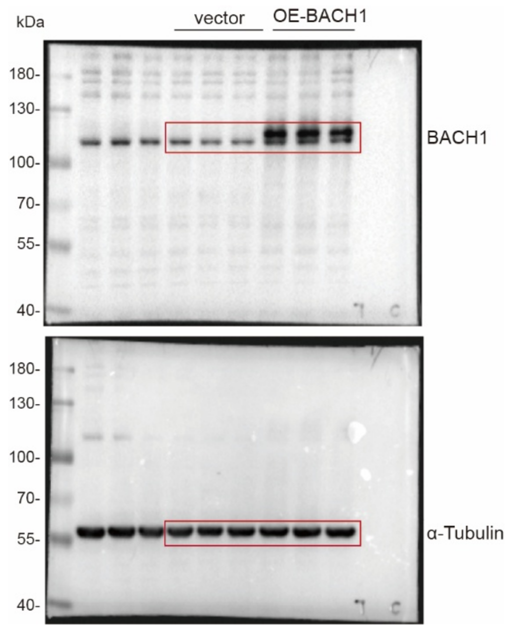

11. Figure S2D: BACH1 knockdown in HTR8/SVneo cells (n=3 per group).

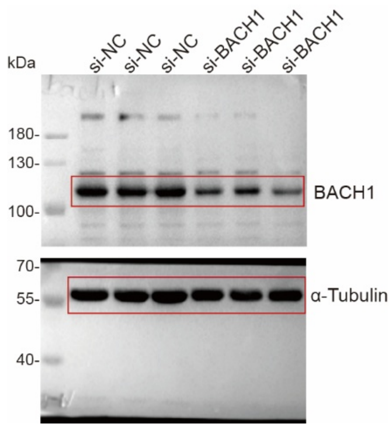

12. Figure S2E: CDK2 in HTR8/SVneo cells (Vector vs. OE-BACH1, n=3 per group).

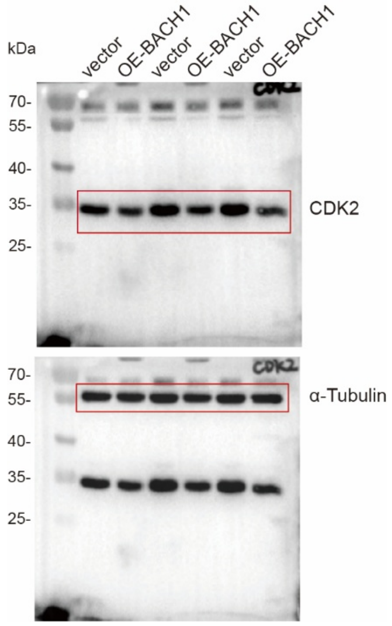

13. Figure S2F: Cyclin A2 in HTR8/SVneo cells (Vector vs. OE-BACH1, n=3 per group).

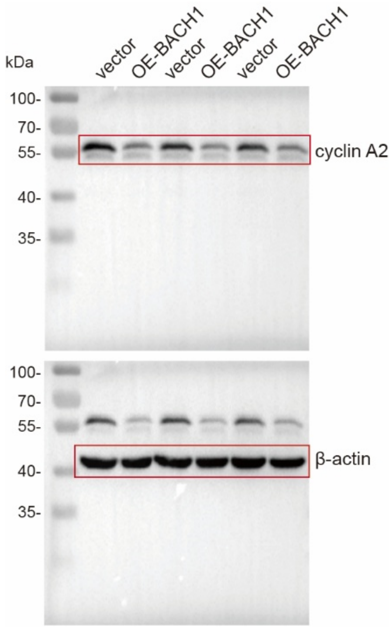

14. Figure S3B: BACH1 overexpression in JEG3 cells (n=3 per group).

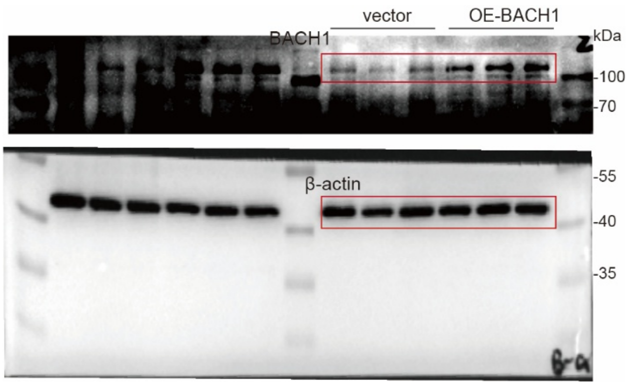

15. Figure S6A: HAT1 in HTR8/SVneo cells (n=3 per group).

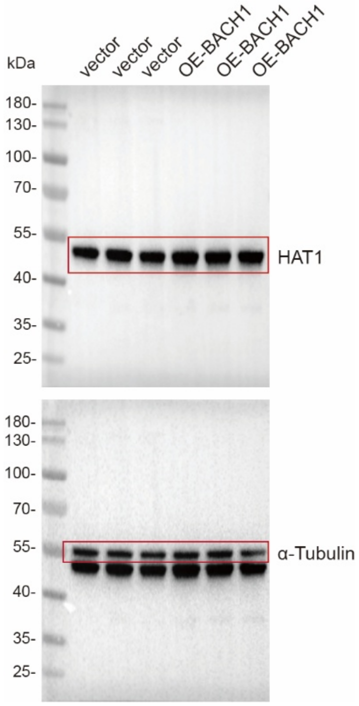

16. Figure S6B: KAT2A in HTR8/SVneo cells (n=3 per group).

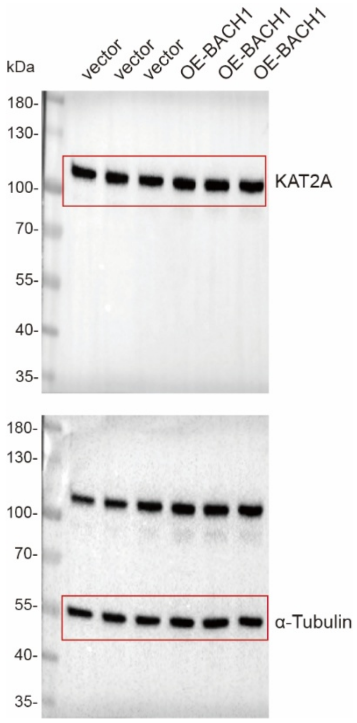

17. Figure S6C: SIRT5 in HTR8/SVneo cells (n=3 per group)

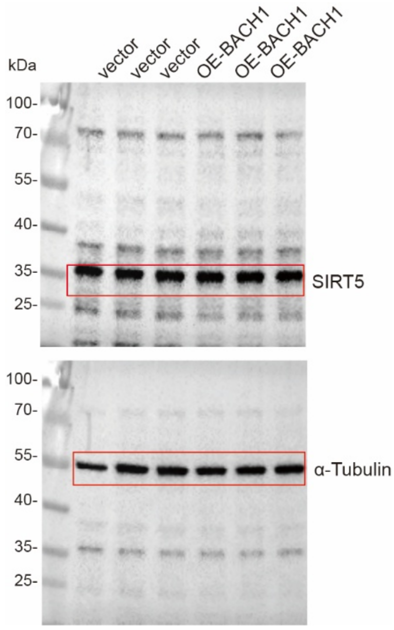

18. Figure S6E: SIRT7 in human placenta (Control n=7, PE n=7).

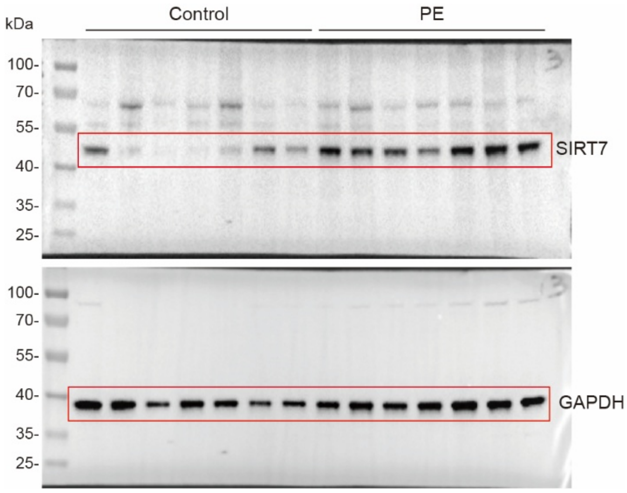

kDa
Control
PE
100-
70-
55-
40-
35-
25-
SIRT7
100-
70-
55-
40-
35-
25-
GAPDH
